# Polymyxins slow down lateral diffusion of proteins and lipopolysaccharide in the *E. coli* outer membrane

**DOI:** 10.1038/s42003-025-09161-x

**Published:** 2025-12-05

**Authors:** Dheeraj Prakaash, Syma Khalid

**Affiliations:** Department of Biochemistry, University of Oxford, Oxford, UK

**Keywords:** Antimicrobials, Biochemistry

## Abstract

Polymyxins are often administered as last resort antibiotics for Gram-negative bacterial infections. However, given unwanted side-effects, efficacy and recent reports of resistance against polymyxins, there is an urgency to develop alternatives. This necessitates an understanding of how polymyxins associate with and translocate across the formidable permeability barrier of the Gram-negative bacterial outer membrane. We employ multi-scale molecular simulations to explore the initial association of polymyxin B1 using *E. coli* outer membrane models that incorporate the latest details of supramolecular lattice networks formed by lipids and native proteins. We show that polymyxin molecules attach to the outer membrane surface, reducing the lateral displacement of proteins and lipopolysaccharides, and polymyxins often associate into large protein-polymyxin aggregates that link individual proteins. Furthermore, we provide atomistic resolution insights into the interaction network between proteins, lipopolysaccharides and polymyxins that lead to the reduced lateral mobility of proteins and lipopolysaccharides in the *E. coli* outer membrane.

## Abstract (web summary)

Multi-scale molecular dynamics simulations reveal that polymyxins form polymyxin-protein aggregates upon associating with the E. coli outer membrane thereby reducing lateral mobility of outer membrane proteins and lipopolysaccharides.

## Introduction

Polymyxins are lipopeptides originally derived from the Gram-positive bacterium *Paenibacillus polymyxa*^1^. They show antimicrobial activity against Gram-negative bacteria, generally thought to be through lytic action on the inner membranes of the pathogens, although this is by no means conclusive. Structurally, polymyxins are composed of a lipophilic tail attached to a cyclical, cationic peptide region, which has a charge of +5 *e*^2^. The two best studied polymyxins are (i) colistin, which is clinically used and referred to as polymyxin E (PME), and (ii) polymyxin B1 (PMB1), which is perhaps most often used in experimental studies. Structurally, they differ only in one residue; PMB1 contains a D-phenylalanine, which is replaced by leucine in colistin.

Despite their relatively poor efficacy and undesirable side-effects of nephro- and neurotoxicity, polymyxins are often administered as last resort antibiotics^3,4^. However, recently there have been reports of the development of resistance to polymyxins, which arises *via* modifications of lipopolysaccharide (LPS) molecules in the outer membrane (OM) of Gram-negative bacteria^3,5^. Specifically, resistance against polymyxins has been reported for *Acinetobacter baumanii*, *Pseudomonas aeruginosa*, and *Klebsiella pneumoniae*^6–8^. Consequently, there is a pressing need to develop alternatives to polymyxins that lack the accompanying side effects. To do so, however, requires a clearer understanding of the molecular mechanism by which polymyxins first associate with and then penetrate the OM.

The theory of the ‘self-promoted uptake’ mechanism of polymyxins^4,9,10^ hypothesizes that association of polymyxins with the OM of Gram-negative bacteria involves binding of the diamino butyric acid (DAB) residues of PMB1 to the anionic moieties of LPS, thereby displacing divalent cations, which non-covalently cross-link LPS molecules. This impacts the structural integrity of the OM when a bulk of polymyxins associate with the OM, and the resultant ‘defect’ enables other polymyxins to insert into the OM. This theory has been probed by both experimental and computational work. Although, experiments have provided insights into the mode of insertion of polymyxins into the OM (or LPS-containing membrane models) using techniques such as (i) confocal imaging^11^, (ii) X-ray scattering combined with circular dichroism^12^, (iii) interfacial tensiometry combined with optical waveguide lightmode spectroscopy, dynamic light scattering and circular dichroism^13^, they do not reveal the dynamic behaviour of polymyxin molecules and their interactions with the OM on a molecular level. On the other hand, molecular dynamics (MD) simulations have been invaluable in providing molecular details of PMB1 insertion into the headgroups of LPS molecules in the OM^14^.

A number of simulation studies of antibiotic insertion into the OM reported in the literature have almost exclusively^15–17^ neglected to include outer membrane proteins (OMPs) that are native to these membranes. We recently reported a molecular model of a portion of the *E. coli* OM, which reveals molecular details of an OMP-dense region constructed from experimental evidence^18^. In this model, the OMPs are positioned such that they satisfy conditions observed from a combination of atomic force microscopy (AFM)^19^, cross-linking, and mass spectrometry data. In this model, the OMPs form a hexagonal lattice. In other recent work, Hiller and co-workers have reported AFM images that reveal a distinct hexagonal lattice of OMPs, particularly after the OM is treated with polymyxins^20^. Given that OMP lattices are prevalent across the surface of the OM, it is our contention that they should be incorporated into any study of mechanisms of insertion into the OM. Here, using multi-scale MD simulations, we have studied the association of two polymyxin types, PMB1 and PMB nonapeptide (PMBN), with our to-scale model of an OMP-dense region. We show that both PMB types form bridges between OMPs, leading to an overall reduction in the lateral displacement of OMPs, and that both preferentially interact with acidic sidechains on OMPs and specific sites on LPS molecules. These interactions, which promote polymyxin association with the OMP lattice, likely also facilitate their penetration into the OM at longer time-scales.

## Results

We performed 10 μs coarse-grained molecular dynamics (CGMD) simulations (in 3 replicates) of our previously reported OM model^18^ which contains an OMP-dense region inserted in a membrane containing ReLPS (outer leaflet) and phospholipids (inner leaflet). The number of PMB1 molecules in our simulation system is 1394 following a 6:1 ratio of LPS:PMB1 molecules as used in a previous study^14^, This number also translates to a PMB1 concentration of 3.4 mM, considering the simulation box dimensions: 150 × 150 x 30 nm^3^, which is consistent with the concentrations of colistin used in dynamic light scattering experiments^21^. All 1394 PMB1 molecules were randomly oriented and positioned along the XY plane i.e., parallel to the membrane surface (Fig. S1A). A similar system containing the same number of PMBN, which lacks the fatty acid tail, was simulated. The PMBs were initially non-aggregated and positioned near the outer leaflet but avoiding contact with the membrane. Once the simulations began, they were observed to rapidly adhere to each other, to ReLPS molecules in the outer leaflet, and to OMPs (while these interactions were not mutually exclusive). The computed average number of PMB1 molecules per aggregate across all replicates was ~ 7, which is comparable with previous atomistic simulations that showed a hexameric assembly of PMB1 molecules^22^. The diameters of PMB1 aggregates before they associated with the OM (at simulation time (t)=1 μs) were 2.60 ± 0.19 nm, comparable to colistin aggregate diameters of 2.07 ± 0.30 nm recorded by light scattering experiments (at colistin concentration >1.5 mM)^21^.

Visual inspection of the final snapshots of each simulation revealed macromolecular aggregates (i.e., OMP-OMP and particularly OMP-PMB-OMP complexes) all over the OMP-dense region. To quantify the number of macromolecular aggregates and their time evolution of aggregate formation, we employed a 2-dimensional image analysis method. Briefly, in this method, taking a bird’s-eye view of the system from the outer leaflet side (Fig. S1B), each individual OMP or aggregate of OMPs was identified and represented by a unique colour (by hex code). Initially, the OMPs were all separated from each other (other than two cases of BamA-BtuB interaction). Thus, 216 different colours were used to represent 218 OMPs at t = 0 ns (Fig. S1C). However, as the simulations proceeded, both PMB types contacted the OM and began mediating OMP-OMP associations forming macromolecular aggregates (a snapshot from t = 10 μs is shown in Fig. S1D). This resulted in fewer ‘uncomplexed’ macromolecules, and therefore the size of distinctly coloured patches in the image (representing each aggregate) increased. This is shown quantitatively *via* a plot of the number of macromolecular aggregates vs time in Fig. S2 and qualitatively using image analysis at different time points in Fig. S3.

In simulations containing PMB1, there were ~ 150 aggregates on average at 1 μs, and this reduced to ~ 130 distinct aggregates at 2 μs, and further to 105 aggregates at 10 μs. A similar trend was observed in simulations containing PMBN, but the average number of aggregates was lower compared to PMB1. Image analysis of snapshots extracted from simulation time t = 0, 1 μs, 2 μs, and 10 μs from each of the PMB1 and PMBN-containing simulations is shown in Fig. S3A, B respectively, along with the number of macromolecular aggregates mentioned below each corresponding image. The simulations revealed large OMP-PMB-OMP aggregates at t = 10 μs, where the OMPs (totally 218) clustered into ~ 105 distinct aggregates in the presence of both PMB types. Additionally, image analysis of these snapshots (t = 10 μs) revealed that the largest macromolecular aggregates in the presence of both PMB types were composed of up to 19 OMPs (Fig. S3A, B).

We further investigated PMB-OMP interaction lifetimes by first categorizing PMB1 and PMBN molecules into (i) those that interacted only with OMPs and did not end up interacting with ReLPS, and (ii) those that did interact with ReLPS and also with OMPs. We quantified their interaction lifetimes by iteratively counting the number of frames that each PMB molecule interacted with an OMP and then measuring the lifetime of each OMP-PMB interaction (Fig. S4). The data revealed that, of those PMBs that did not interact with ReLPS, PMBN-OMP contacts exhibited longer lifetimes compared to PMB1-OMP contacts (shown for each simulation replicate in Fig. S4A). Data from all 3 replicates (10 μs each) combined showed that these PMBN molecules remained bound to OMPs for long time periods (9–10 μs, Fig. S4B), whereas PMB1 showed both long (9–10 μs) and short interaction lifetimes (0–1 μs), but predominantly the latter (Fig. S4B). In comparison, the PMBs that did interact with ReLPS were able to form relatively transient interactions (mostly ranging from 0 to 1 μs) with OMPs (Fig. S4C, D); the contacts vs time data for these PMBs shows more frequent association and dissociation events (shown as coloured and white heatmap cells in Fig. S4C for each simulation replicate). Data from all 3 simulations combined shows that the distribution of PMB-OMP interaction lifetimes between OM-inserted PMB1 and PMBN overlapped, where there were predominantly short interaction lifetimes (0–1 μs, Fig. S4D). This suggested negligible difference between the interaction dynamics of the two PMB types with OMPs once they were associated with ReLPS. In summary, the distributions of PMB-OMP interaction lifetimes highlighted short-lived and long-lived PMB-OMP interactions depending on the PMB type when they did not interact with ReLPS, and predominantly short-lived interactions when they did interact with ReLPS, irrespective of PMB type.

### PMBs form bridges that link neighbouring OMPs

**Fig. 1:**
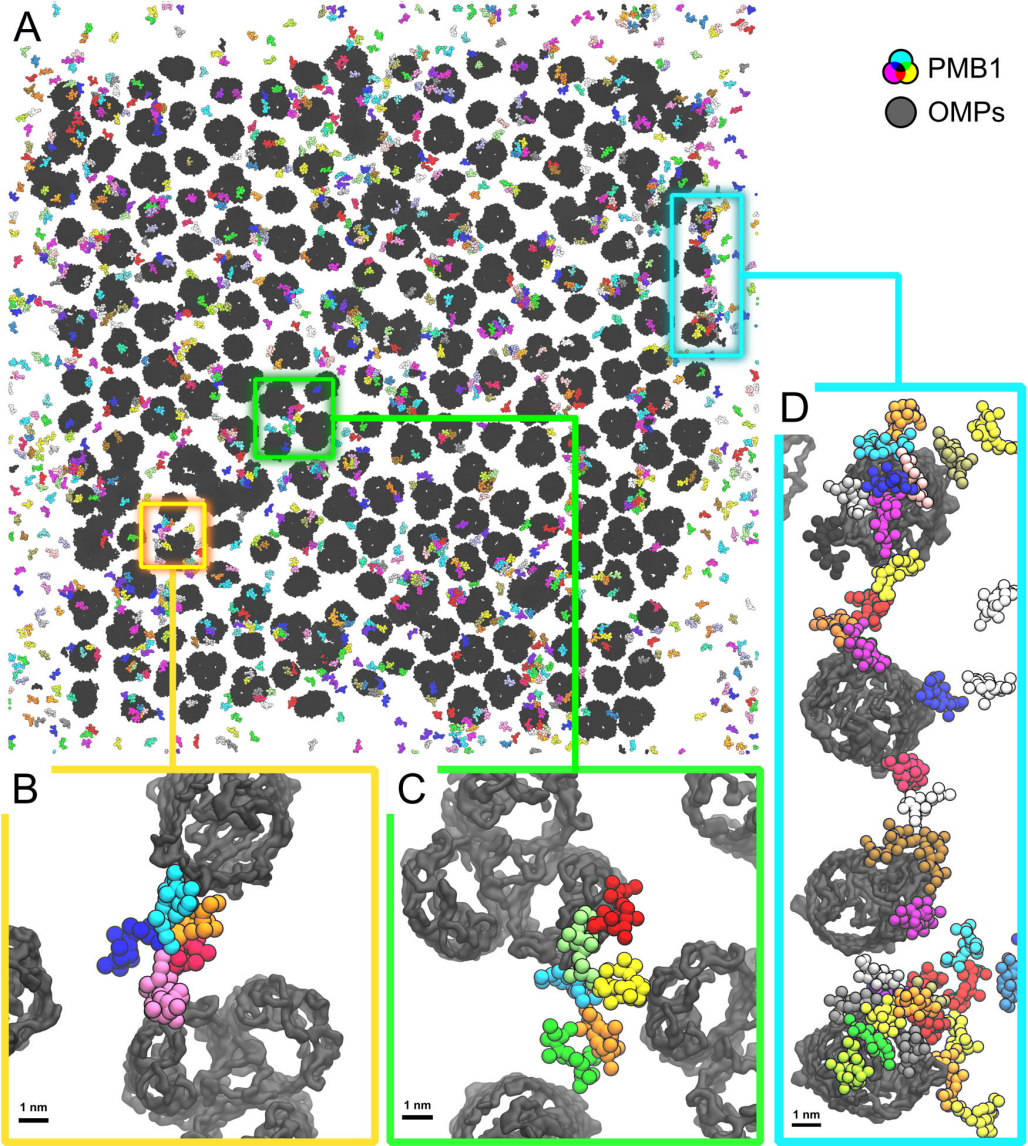
PMB-mediated OMP bridges. **A** Top view (from the exterior side) of one of the CGMD simulations containing PMB1 at t = 2 μs. OMPs are shown in black, and PMB1 molecules are coloured by individual molecule. **B** Magnified top-down views of PMB1 molecules bridging 2 OMPs, (**C**) 3 OMPs, and (**D**) 4 OMPs.

Visual inspection of the macromolecular aggregates from our CGMD simulations of an OMP-dense region at t = 2 μs (one simulation snapshot shown in Fig. 1A) revealed that, in addition to a few instances of PMBs interacting with an isolated OMP, there were more often PMB ‘bridges’ observed. These bridges can be broadly described as (i) one or more PMBs interacting simultaneously with two OMPs (Fig. 1B), or (ii) multiple PMB molecules connecting 3 or more OMPs (Fig. 1C, D).

**Fig. 2:**
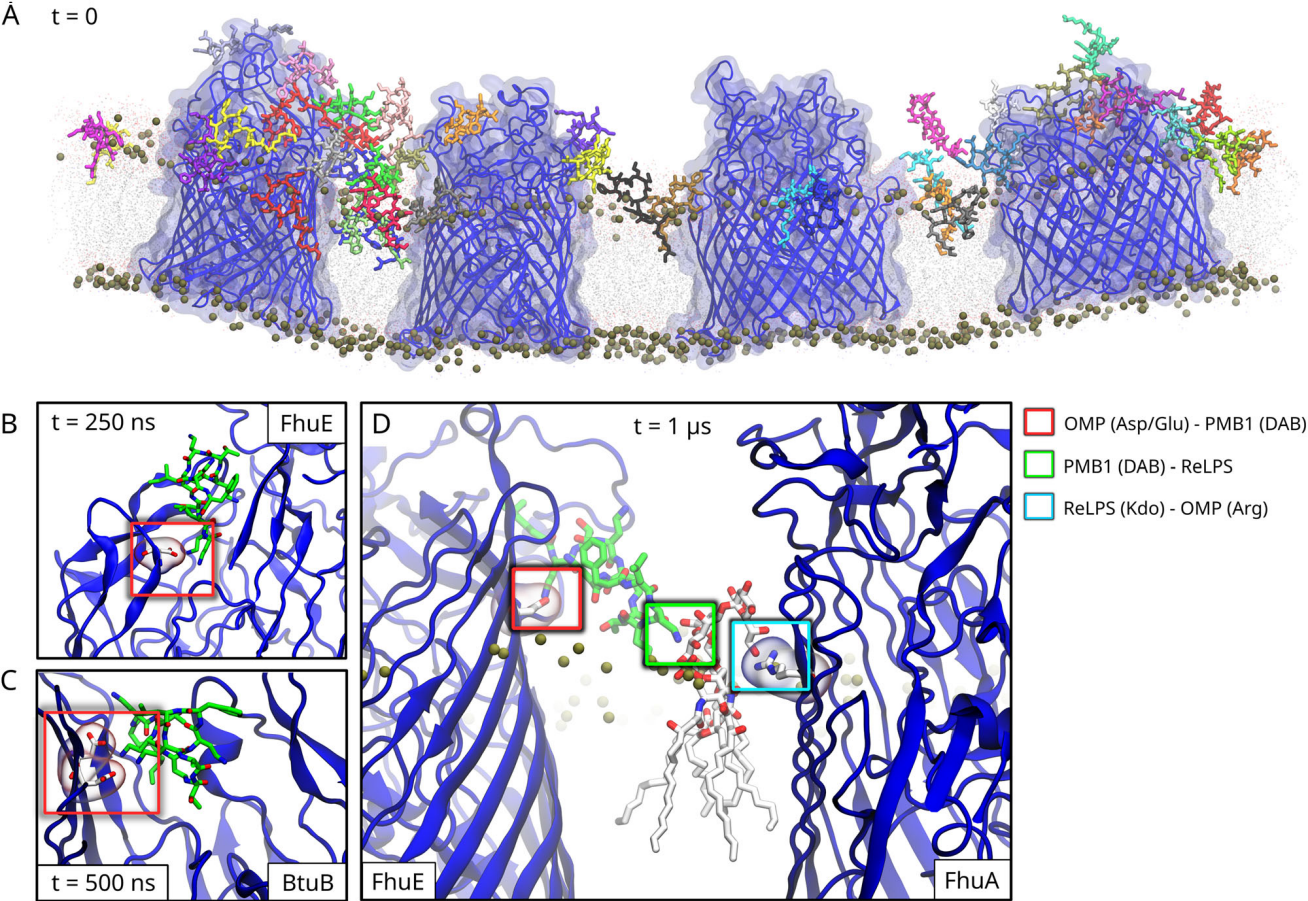
OMP-PMB1 bridge in atomistic resolution. **A** A snapshot of a PMB1-mediated bridge between 4 OMPs from one of the atomistic simulations (t = 2 μs). In this snapshot, the OMP backbones are shown as blue cartoon and the surface of the OMPs are shown in transparent blue. Each PMB1 molecule is coloured distinctly. The lipid phosphate headgroups are shown as brown spheres. Other ReLPS molecules, phospholipids, water, and ions are hidden for visual clarity. **B** Electrostatic interactions between the DAB sidechains of a PMB1 molecule and an aspartate residue of an OMP at t = 250 ns and (**C**) t = 500 ns. **D** An OMP-PMB1-LPS-OMP bridge. At t = 1 μs, we observe a PMB1 molecule forming an electrostatic interaction with an aspartate (of FhuE) and with the Kdo sugar moiety of a ReLPS molecule simultaneously. This ReLPS, while interacting with the PMB1, was found forming an electrostatic interaction with an arginine residue of a different OMP (FhuA).

Atomistic simulations were next employed to characterize PMB1-OMP interactions in more fine-grained chemical detail. Conversion and subsequent simulation of our entire CG system to atomistic detail would be prohibitively expensive for anything other than very short simulations. Consequently, we backmapped only certain regions of our CG systems into atomistic resolution (as highlighted in Fig. S5A) using the following criteria: (i) regions with high number densities of PMB1 to promote sampling of PMB1-OMP interactions, and (ii) regions revealing bridges of PMB1 molecules linking neighbouring OMPs (as shown in Fig. 2A). Each atomistic system was simulated for 1 μs in 3 replicates each (i.e., total of 3 μs for each system). The overall positions of the OMPs and associated PMB1 molecules with respect to each other were maintained until the end of the simulations, although in some cases the OMPs, PMBs, and ReLPS molecules were displaced laterally as a single entity (Fig. S5B). Although the aggregates remained intact overall, OMP-PMB contacts measured at t = 0, 250, 500, 750, and 1000 ns revealed local dissociations and associations between neighbouring OMPs and PMB1 molecules across all atomistic systems (Fig. S5C). Overall, at the end of 12 out of 15 atomistic simulations (5 systems x 3 simulation replicates), we noted a decrease in the number of uncomplexed macromolecules i.e., an increase in the number of PMBs forming bridges between OMPs.

A number of interactions between PMB1 and both OMPs and ReLPS were observed. An example is electrostatic interactions between the DAB sidechains of PMB1 molecules and the acidic (Asp / Glu) amino acid sidechains of OMPs, which were observed in multiple systems and at multiple time points (two snapshots from a single system are shown in Fig. 2B, C). To quantify these interactions of PMB1 with OMPs and ReLPS in all atomistic simulations (5 systems x 3 simulation replicates), we computed (i) the number of hydrogen bonds formed by polar regions of PMB1 with charged/polar amino acid sidechains (Fig. S6A) and with moieties of ReLPS molecules (Fig. S6B), and (ii) hydrophobic contacts formed by apolar regions of PMB1 with apolar amino acid sidechains (Fig. S6C) and with moieties of ReLPS molecules (Fig. S6D). The data revealed that the PMB1 DAB sidechains preferred to electrostatically interact with the sidechains of aspartate residues followed by glutamate residues on OMPs (Fig. S6A), while the predominant electrostatic PMB1-ReLPS interactions were between the PMB1 DAB sidechains and the ReLPS Kdo sugars (Fig. S6B). On the other hand, the most number of hydrophobic interactions of PMB1 molecules were formed *via* their hydrophobic tails with proline and tryptophan sidechains (Fig. S6C) and with the fatty acid tails of ReLPS (Fig. S6D).

**Fig. 3:**
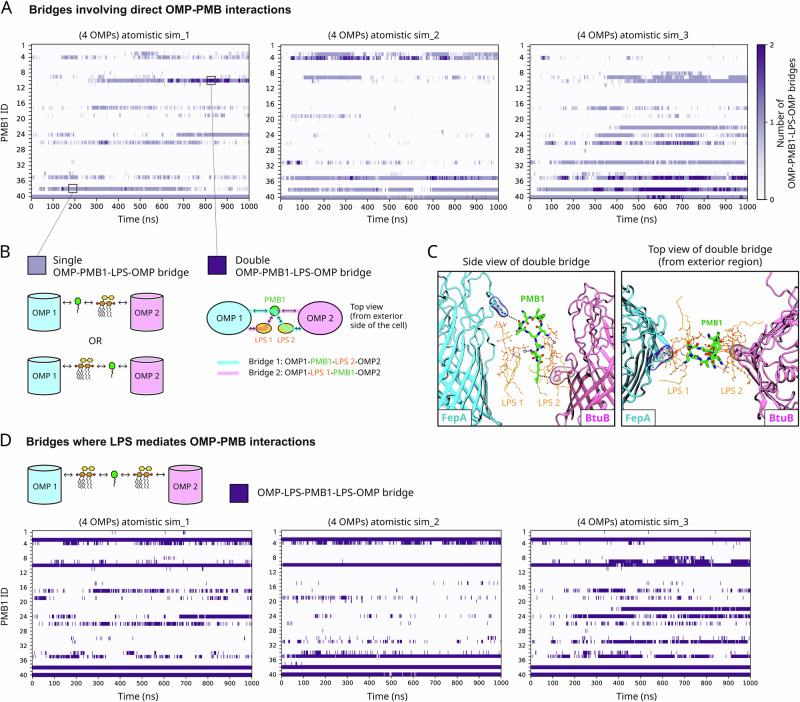
OMP-PMB1-LPS-OMP and OMP-LPS-PMB1-LPS-OMP bridges in atomistic resolution. **A** Number of OMP-PMB1-LPS-OMP bridges vs time data for each simulation replicate of atomistic system-1 (the one containing 4 OMPs). The Y-axis lists each PMB1 molecule in the simulation, and the depth of the color of the bar indicates single (light purple) or double bridge (dark purple). Data for atomistic systems 2–5 is shown in Fig. S7A. **B** Schematic illustration of single and double OMP-PMB1-LPS-OMP bridges. **C** Side view (along the plane of the membrane; left) and top view (from the extracellular region; right) of a snapshot of a double bridge, where a PMB1 molecule interacts directly with 2 OMPs, FepA and BtuB, and also with 2 LPS molecules, each interacting with one of these OMPs. For comparison, a snapshot of a single bridge is shown in Fig. 2D. OMP backbones are shown in blue cartoon representation, PMB1 as thick green sticks, and ReLPS as thin gray sticks. Amino acids interacting with the PMB1 are highlighted using their surface representations. Hydrogens are hidden for visual clarity. The bridges referred to in panels (**A**–**C**) involve direct OMP-PMB interactions. **D** Number of OMPs in OMP-LPS-PMB1-LPS-OMP bridges vs time data for each simulation replicate of atomistic system-1. These bridges do not involve direct OMP-PMB interactions but are mediated by LPS molecules. Data for atomistic systems 2–5 is shown in Fig. S9A.

We also computed the number of hydrogen bonds formed by each PMB1 molecule with its neighbouring OMP and ReLPS molecules simultaneously (across all 15 simulations i.e., 5 systems x 3 simulation replicates; Fig. S7). In all simulations, we quantitatively determined that each PMB1 molecule had simultaneously contacted an OMP and ≥ 1 ReLPS molecule(s) at least once by the end of the simulation (1 μs). Moreover, we noted that these PMB1 molecules bridged OMP-ReLPS interactions at various time points in each simulation, totalling to 18% of all frames across 15 simulations. This included cases of PMB1 molecules bridging an OMP to an ReLPS molecule, which was engaged in an interaction with a different, neighbouring OMP. For clarity, an example of this is shown in Fig. 2D, whereby a PMB1 is engaged simultaneously in hydrogen bonds with (i) an aspartate residue on the surface of a FhuE protein, and (ii) the Kdo sugar of an ReLPS molecule, while this ReLPS is engaged in a hydrogen bond with an arginine residue of a FhuA protein. So, the pattern of interactions here is FhuE-PMB1-ReLPS-FhuA, where the hyphens indicate a direct interaction. We extracted time-series data for all occurrences of such bridges from all atomistic simulations. The data for the first atomistic simulation system is shown in Fig. 3A, and the remaining systems are shown in Fig. S8A (for visual reference of all atomistic systems, their snapshots at t = 0 and t = 1 μs are shown in Fig. S5B). This time-series data revealed ‘single’, ‘double’, and ‘quadruple’ bridges. These are defined as follows: In a single OMP-PMB1-LPS-OMP bridge (as observed in Fig. 2D), a single PMB1 mediates an interaction between an OMP and an ReLPS molecule, where the latter is also in contact with another OMP. In a double OMP-PMB1-LPS-OMP bridge, the PMB1 forms 2 different bridges *via* different combinations of OMP-PMB1-LPS-OMP molecules (schematically illustrated in Fig. 3B and a snapshot shown in Fig. 3C). In a quadruple bridge, the PMB1 interacts with a group of 3 OMPs and 3 ReLPS molecules *via* 4 bridges in arrangements that are schematically illustrated in Fig. S8B.

The role of LPS in these bridging interactions was further highlighted in observations of OMP-LPS-PMB1-LPS-OMP bridges where 2 or more ReLPS molecules, each contacting different OMPs, form interactions with a common PMB1 molecule (schematically illustrated in Fig. 3D and a simulation snapshot shown in Fig. S9). We obtained time-series data for these bridges as well (data for each individual simulation of the first atomistic system are shown as heatmaps in Fig. 3D and the remaining systems are shown in Fig. S10A). These data revealed that ReLPS can mediate interactions with PMB1 molecules such that a single PMB1 is bridged to up to 4 OMPs each *via* a different ReLPS molecule (schematically illustrated in Fig. S10B and a snapshot shown in Fig. S10C). Thus, units composed of up to 4 ReLPS, 4 OMPs, and 1 PMB1 emerge.

Overall, we infer that PMB1 molecules tend to form interactions simultaneously with OMPs and ReLPS molecules in their vicinity in various stoichiometries and molecular arrangements.

Recent experimental (AFM, cross-linking, and mass spectrometry) data has shown that the *E. coli* surface is separated into (i) regions densely populated with OMPs^19^, in which the OMPs are arranged into irregular hexagonal lattices with at least one shell of LPS molecules around each OMP^18^, and (ii) smaller regions of LPS from which OMPs are excluded^19^. It has been suggested by other AFM studies that polymyxins may play a role in inducing hexagonal arrays of OMPs on the *E. coli* surface^20^. Given that our simulations reveal networks of interactions between PMB1, ReLPS, and OMPs (as shown in Figs. 2D, 3B–D, S8–S10), we next sought to determine the impact (if any) of PMBs on the organization and mobility of OM components.

### Effect of PMBs on the lateral mobility of OM components

**Fig. 4:**
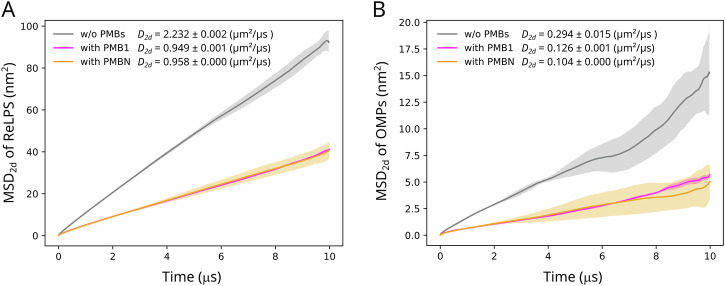
Impact of PMB1 and PMBN on the lateral displacement of OMPs and ReLPS. **A** Averaged lateral MSD vs time of ReLPS and (**B**) OMPs, are calculated from 3 simulation replicates for 3 systems each i.e., simulations containing (i) no PMB1/PMBN (grey), (ii) PMB1 (magenta), and (iii) PMBN (orange). Averaged data curves are shown as lines while their standard deviations are shown as transparent shaded regions of the same colour. Lateral diffusion coefficients (*D*_*2d*_) of ReLPS and OMPs calculated using their averaged MSD_2d_ values for each system are mentioned in the respective plots.

To determine the impact of the PMB association with the OM on the lateral mobility of OMPs and ReLPS, we analyzed the lateral mean squared displacement (MSD_2d_) of OMPs and ReLPS molecules (i.e., along 2 dimensions – the plane of the membrane) in the CGMD simulations of the OMP-dense region. The OM model was also simulated for the same timescale without PMBs to provide a baseline for comparison. We found that, in all 3 simulations, both PMB types, when associated with the OM resulted in a marked reduction of the lateral displacement of ReLPS (Fig. 4A) and OMPs (Fig. 4B). We calculated the lateral diffusion coefficients using their averaged MSD_2d_ values. The lateral diffusion coefficient (*D*_*2d*_) of ReLPS molecules in presence of PMB1, PMBN, and in the absence of PMBs obtained were 0.949 ± 0.001, 0.958 ± 0.00, and 2.232 ± 0.002 μm^2^/μs respectively. Given that we are using a model of ReLPS with enhanced kinetics^23^, comparing diffusion coefficients calculated only from the three systems we have simulated is key, rather than noting their absolute values. We performed the same calculation for OMPs and obtained *D*_*2d*_ = 0.126 ± 0.001, 0.104 ± 0.00, and 0.294 ± 0.015 μm^2^/μs in the presence of PMB1, PMBN, and in the absence of PMBs, respectively. We infer from these calculations that the reduction in the lateral diffusion of ReLPS induced by PMB1 and PMBN was very similar i.e., PMB1 and PMBN-induced reduction in ReLPS lateral diffusion were reduced by a factor of 2.35x and 2.33x respectively compared to that in the absence of PMBs. Similarly, the lateral diffusion of OMPs due to PMB1 and PMBN was reduced by a factor of 2.33x and 2.82x respectively compared to that in the absence of PMBs. On the other hand, we found that the lateral displacement of phospholipids in the inner leaflet of the OM was not affected by the presence of PMBs (Fig. S11).

Given that the lateral diffusion coefficient of OMPs was reduced by a factor of ~ 2 as calculated above, it is unsurprising that our CGMD simulations revealed that the near-neighbour distance distribution (NNDD) of OMPs associated with both PMB types was consistent with the overall NNDD in the native *E. coli* OM calculated from AFM experiments^19^ and our own previous simulations^23^ (neither of which included polymyxins) (Fig. S12). Therefore, we deduced that the association of polymyxins further restricts the lateral motion of ReLPS and OMPs. Moreover, our NNDD data is consistent with both sets of AFM data i.e., those obtained in the presence and absence of polymyxins^24^ suggesting no major alteration to the positions of the OMPs upon incubation with polymyxins. However, the resolution of AFM images obtained before and after incubation with polymyxins (colistin) showed a significant difference^20^, with clearly defined crystalline hexagonal lattices of OMPs visible after polymyxin incubation compared to lower resolution images in the absence of polymyxins. Therefore, we posit here that it is the reduction in the lateral diffusion of the OMPs and ReLPS that enables higher resolution AFM data when polymyxins attach to the OM. In our simulations, both PMB types acted like clamps that retarded the lateral motion of the OM components.

Interestingly, further analysis revealed that the ReLPS molecules within the OMP-dense region (mostly < 2 nm from an OMP surface) exhibited reduced lateral motion compared to ReLPS molecules outside this region ( > 2 nm of OMPs). While this trend in ReLPS diffusion was the same irrespective of the presence of PMBs, where the presence of just OMPs restricted ReLPS motion, the combined impact of both OMPs and PMBs resulted in the most restricted ReLPS lateral motion within the OMP-dense region when PMBs are present (Fig. S13). We also identified some correlation in the directionality of flow of ReLPS molecules within the OMP-dense region (Fig. S14A) as previously seen even with systems containing less density of OMPs^25^. Interestingly, the flow analysis indicated less correlation outside the region of high OMP density, and these regions also tend to have greater density of PMBs interacting directly with ReLPS (Fig. S15). On the other hand, the flow of phospholipids in the inner leaflet revealed no such correlation in their flow directionality in any region of the membrane (Fig. S14B). Given this impact of PMBs on reducing molecular mobility in the outer leaflet of the OM, we were intrigued as to how PMBs inserted into the outer leaflet of the OM and thus investigated this next.

### Replacement of cations by DAB sidechains of PMBs

**Fig. 5:**
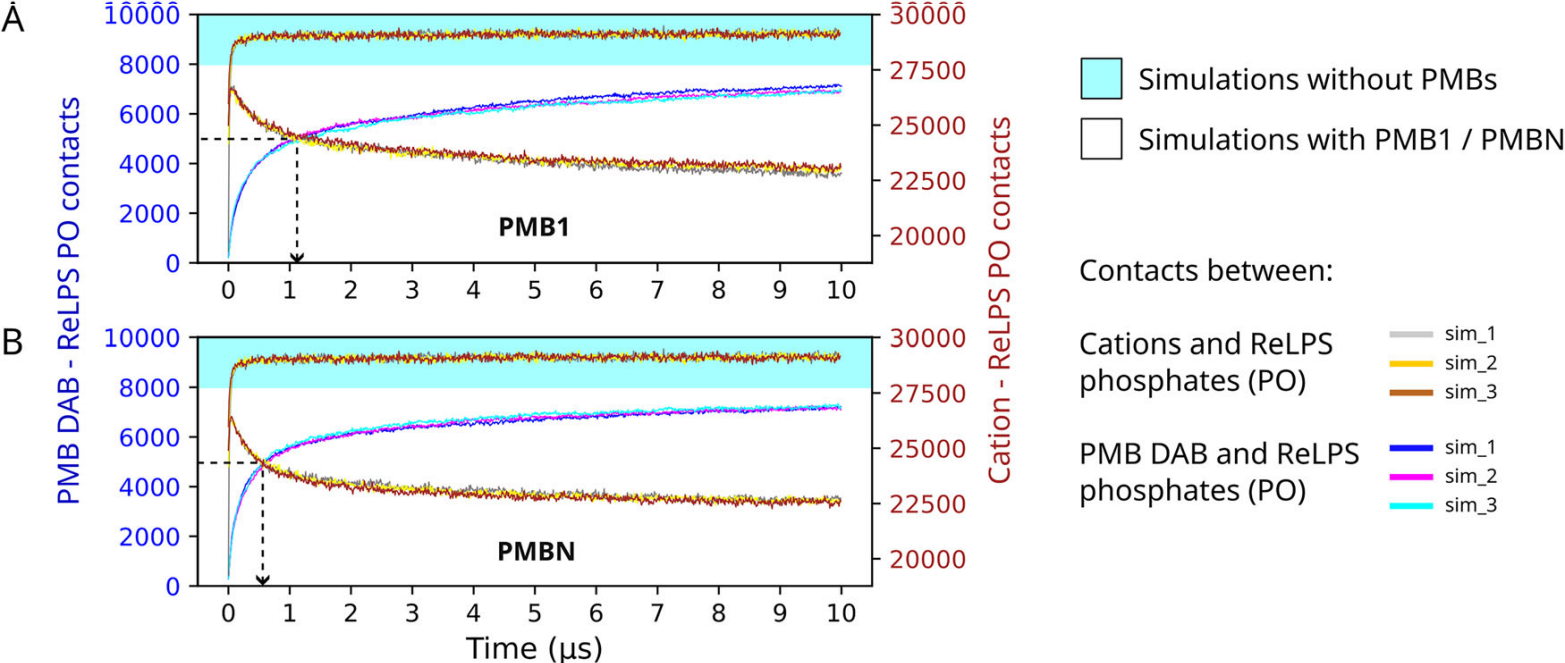
Replacement of cations by DAB sidechains of PMBs. The number of cation-ReLPS phosphate and DAB-ReLPS phosphate contacts vs time from all 3 CGMD simulation replicates are shown individually for (**A**) PMB1 and (**B**) PMBN-containing systems, each compared to those from simulations without PMBs. The number of contacts for (i) PMB DAB sidechains - ReLPS phosphates (PO) are shown as blue text on the left Y axis, and (ii) cation- ReLPS PO are shown as brown text on the right Y axis. Note that the Y axes shown on either side of the plots are on different scales. The dotted line arrows indicate that the DAB–ReLPS PO contacts rise and the cation–ReLPS PO contacts drop within a shorter period of time in PMBN simulations compared to PMB1 simulations. The cation – ReLPS PO contacts data from simulations without PMBs (cyan shaded region) is plotted for reference.

While there is lot of experimental evidence for the insertion of PMB1 into the OM of Gram-negative bacteria^4,5,11,26,27^, the mechanism of insertion is less well understood. It has been hypothesized that the first step of insertion proceeds *via* the charged DAB moieties of polymyxins, replacing the cations that cross-link the phosphate groups of lipid A^3,4,9–11^. We first monitored the number of interactions between cations and ReLPS phosphate groups as a function of time in simulations without PMBs. The number of contacts rapidly increased at the beginning of the simulations as the cations settled between ReLPS molecules but plateaued within 200 ns remaining stable thereafter for the remainder of the 10 μs simulations. In contrast, in the presence of both PMB1 and PMBN, while we observed a similar initial increase in the number of cation-ReLPS phosphates interactions, these peaked at ~50 ns (with the number of interactions almost 5% lower than in the absence of PMBs at that time point) after which the number of interactions slowly decreased throughout the simulations (Fig. 5A, B). Concurrently, the PMB DAB-ReLPS interactions increased steadily throughout the simulations. At the end of the 10 μs simulations, the number of cation-ReLPS interactions in systems containing PMB1 and PMBN was 21% and 22% lower than in simulations without PMBs, respectively. Similar behaviours were noted for both PMB1 (Fig. 5A) and PMBN (Fig. 5B), providing more evidence for the notion that it is the DAB residues of PMBs that lead to the displacement of cations.

Furthermore, we observed that the number of DAB residues of PMB1 contacting ReLPS phosphates (*n*) reached a certain magnitude (*n* = 5000) at a later time point ( ~ 1.1 μs) compared to DAB residues of PMBN ( ~ 0.6 μs to reach *n* = 5000; indicated by dotted line arrows in Fig. 5A, B). This time-delay in PMB1 molecules associating with the OM is due to their tendency to form aggregates in solution driven by hydrophobic interactions between their fatty acid tails. We validated this by calculating the change in number of PMB1 and PMBN aggregates through time (Fig. S16) revealing that PMBN molecules dissociated into individual molecules at an earlier time point compared to PMB1.

### Insertion of PMBs into the ReLPS leaflet of the OM

Following the PMB DAB sidechains replacing cations to interact with ReLPS phosphates, we observed penetration of both PMB1 and PMBN beyond the ReLPS phosphate headgroups. In our previous work, we had observed insertion of PMBs into an OM model in the absence of OMPs^23^, which is a good model for OMP-free regions that we now know exist in the *E. coli* OM based on AFM studies^19^. Here, we were interested in elucidating the molecular details of the mechanism of PMB insertion into OMP-rich regions.

**Fig. 6:**
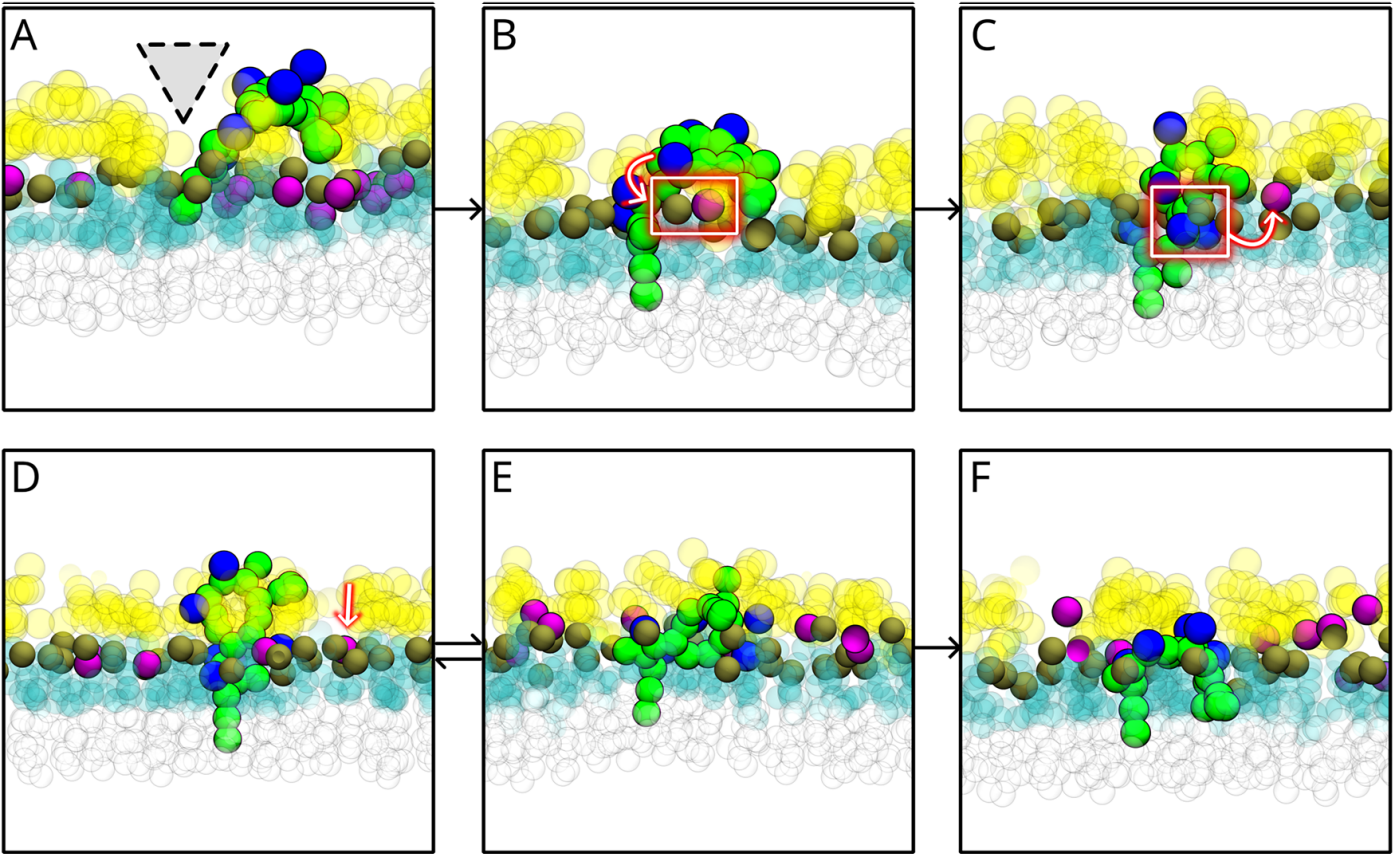
Insertion mechanism of PMB1 into the ReLPS leaflet. **A** PMB1 interacts initially with the Kdo sugar moieties of ReLPS molecules, and the fatty acid tail of PMB1 inserts into the hydrophobic region of the OM through a crevice (marked as a gray triangle) in the Kdo region of ReLPS molecules. **B** A phosphate headgroup of an ReLPS molecule interacting with a cation. **C** One of the PMB1 DAB residue sidechains replaces the cation to interact with the phosphate headgroup. In (**B,** **C**), other cations are hidden for visual clarity. **D** The elongated conformation, (**E**) L-shaped conformation, and (**F**) inverted U-shape conformation of the PMB1 molecule. Note that the double-sided arrow between (**D,** **E**) panels indicates that the PMB1 molecule can switch between these (‘elongated’ and ‘L-shaped’) conformations. The cation replaced by a DAB sidechain in (**C**) interacts with a phosphate group of a different ReLPS molecule in (**D**). ReLPS Kdo sugar moieties are coloured in transparent yellow, ReLPS phosphates in opaque brown, OH/NH-linked tails of ReLPS in transparent blue, hydrocarbon chain of ReLPS tails in transparent white, cations in opaque magenta, PMB1 molecule in opaque green, and its DAB sidechains are in dark blue.

In the presence of OMPs, we commonly observed two insertion mechanisms of PMB1 and PMBN molecules: (i) they first form electrostatic (DAB-Asp, DAB-Glu) interactions with OMPs before interacting with ReLPS molecules (Fig. S17A), and (ii) they directly interact with ReLPS molecules in OMP-free regions (Fig. S17B) regardless of whether they existed previously as an aggregate or an individual molecule. Nonetheless, both insertion mechanisms led to PMB1/PMBN molecules to first interact with the Kdo sugar moieties of ReLPS followed by the insertion of fatty acid tails of PMB1 molecules into regions that were devoid of ReLPS Kdo sugars and phosphates (Fig. 6A). These regions, referred to as ‘crevices’, were a result of tilting of ReLPS molecules and inter-molecular association of Kdo sugars between neighbouring ReLPS molecules. Note that an expanded version of Fig. 6 which includes a view of these crevices from the extracellular region is provided in the supplementary information (Fig. S18). We also observed some PMB1 molecules insert their phenylalanine side chains into these crevices. PMBN insertion always proceeded *via* phenylalanine sidechain insertio,n given the lack of fatty acid tails. The location of the crevices changed dynamically (as frequently as every 20 ns in our simulations, shown by regions shaded in gray in Fig. S18C). Nonetheless, the insertion of the PMB fatty acid tails into the hydrophobic core of the OM resulted in the movement of DAB residues toward the plane of the ReLPS headgroups. This led to the interaction of DAB residues with the ReLPS phosphates by replacing cations (Fig. 6B, C) as previously discussed. At this stage, the hydrophobic amino acid sidechains of PMB1 remained in the plane of the Kdo sugars of ReLPS inducing a conformational switch between the ‘elongated’ (Fig. 6D) and ‘L-shaped’ conformations of PMB1 (Fig. 6E). This re-orientation occurred over a range of timescales dependent upon the local environmental conditions of the PMB. For example, DAB-Kdo interactions (particularly DAB sidechain beads B13 and B15 interacting with the S10 anionic bead in the Kdo region of ReLPS) promoted the elongated conformation of PMB1 (Fig. S19A) whereas DAB-phosphate (*via* DAB sidechains B13 and B15) interactions promoted its L-shaped conformation (Fig. S19B). Finally, from their ‘L-shaped’ conformation, PMB1 molecules inserted their phenylalanine and leucine sidechains into the hydrophobic region of the OM adopting an ‘inverted U-shaped’ conformation (Fig. 6F). These conformations have been observed in previous simulation studies at both coarse-grained and atomistic resolutions^14,28–30^.

To characterize PMB interaction patterns with ReLPS after insertion, we measured the number of contacts between all beads of PMB1/PMBN molecules that inserted into the membrane and each bead of the lipid A region of ReLPS molecules. Our contact analyses showed that both PMB1 (Fig. S20A) and PMBN molecules (Fig. S20B) consistently interacted most with the two phosphate beads (PO1 and PO2) of ReLPS, particularly the PO2 bead, which is the closest phosphate to the OM outer leaflet surface (Fig. S20C). The next highest number of interactions was with the glucosamine (GlcN) sugar moieties (‘GM’ beads) in the lipid A region, followed by the OH/NH-linked regions of their fatty acid tails (‘GL’ beads), the fewest number of interactions were with the alkyl chains of the fatty acid tails (‘C’ beads). Interestingly, there is a marked preference for interaction of both PMBs with GL5 and GL7 beads (indicated with arrows in Fig. S20A–C) compared to other GL beads. The GL5 and GL7 beads are located in the fatty acid tails, which stem from the sugar moiety (GM 4-6 beads) that is connected to the PO2 bead. Therefore, this region of the lipid A comprising PO2, GL5, and GL7 beads may define a binding site for OM-inserted PMBs (highlighted in a gray quadrilateral region in Fig. S20C). Simultaneous interactions of some PMB1 molecules with both PO1 and PO2 phosphate beads of the same ReLPS molecule were also observed (Fig. S20D shows a single PMB1 interacting with both phosphates of LPS 2), likely enabled by the close proximity of the phosphates to each other. There were fewer interactions with GL6 and GL8 as they are located deeper in the hydrophobic region of the OM (Fig. S20C) where there is less PMB penetration in our simulations.

Lastly, despite our data showing that PMBs interacted mostly with the polar components of ReLPS molecules and least with the hydrophobic region (‘C’ beads) of their fatty acid tails, we recorded 920.66 ± 5.86 PMB1 and 975.66 ± 2.89 PMBN molecules (out of 1394 each) that inserted into the hydrophobic core of the OM at the end of the 10 μs CGMD simulations. This constituted 66% and 70% of all PMB1 and PMBN molecules in their respective simulation systems and are likely the largest fraction of PMB molecules to spontaneously insert into the hydrophobic core of the OM in any simulation study so far without the need of enhanced sampling methods.

## Discussion

In this study, we characterize PMB insertion into the *E. coli* OM using a molecular model of the OM which incorporates LPS and OMPs, and where OMPs are arranged in a lattice as revealed by super-resolution fluorescence microscopy data^19^. The number of PMBs simulated in our study translates to a concentration of 3.4 mM, which is consistent with the concentrations of colistin used in dynamic light scattering experiments^21^. Our simulations revealed molecular details that advance understanding of the impact of PMBs on the lateral diffusion of OMPs and LPS molecules, and the mechanisms by which PMBs may insert into the outer leaflet of the OM.

Previously, high-speed AFM studies had shown that images of the *E. coli* OM vesicles after incubation with polymyxin gave rise to visually clear images of crystalline hexagonal OMP lattices compared to images obtained in the absence of polymyxins^20^. Our CGMD simulations revealed that PMBs formed bridges to mediate OMP-OMP aggregates involving ReLPS in various patterns and stoichiometries, and this led to reduced lateral diffusion of both OMPs and ReLPS molecules. Further, conversion of these bridges from CG to AT resolution enabled finer-grained characterization of the interactions, which are largely electrostatic in nature. We infer that it is the reduction in the mobility of the OM components that likely enables higher resolution AFM images when *E. coli* samples are incubated with polymyxins. Moreover, biochemical cross-linking experiments have shown that LPS mediates OMP-OMP associations within the OMP-dense regions^18^ of the OM, where the lateral diffusion and directionality of the flow of LPS within these OMP-dense regions is likely restricted and influenced by that of OMPs. While a similar phenomenon has been observed in a previous simulation study (from our own group) in the absence of polymyxins^25^, here the spatial organization and density of OMPs enable a more realistic characterization. The impact of the polymyxins on ReLPS flow is difficult to quantify, given that, even in systems as large as those we have simulated here, there are relatively few occurrences of polymyxin insertion directly into the ReLPS-rich regions. However, visual inspection suggests less correlated motion where these regions do exist. Nonetheless, a more extensive study is needed to fully explore this potential phenomenon.

The ‘self-promoted uptake’ mechanism hypothesizes that divalent cations that non-covalently link neighbouring LPS molecules are displaced by an initial group of polymyxins, thereby loosening the OM and increasing its permeability to other polymyxin molecules^4,9,10^. Here, we show direct evidence for competition between cations and PMBs, providing support for this hypothesis. However, our simulations do show a slight divergence from the classical ‘self-promoted uptake’ theory, which suggests that the insertion of PMBs into the OM is initiated by the interaction of DAB residues with LPS phosphates^4^. In contrast, in our simulations, it was uncommon for the DAB residues of PMBs to directly contact the phosphates of ReLPS molecules due to the biophysical barrier formed by the Kdo sugars of ReLPS, as observed in previous simulation studies^14,23,31^. Instead, it was the fatty acid tail or hydrophobic residues of PMBs that first inserted into the hydrophobic region of the OM driving the DAB residues towards the ReLPS phosphates. This scenario is likely in native Gram-negative OMs containing (a mixture of) LPS species with different oligosaccharide chain lengths, in which longer oligosaccharides would act as a biophysical barrier against polymyxins. Consequently, for entry into the outer leaflet, polymyxins would first have to insert their fatty acid tail or hydrophobic sidechains into regions of the OM with lower density of oligosaccharides or shorter LPS molecules to access the phosphates of LPS molecules. What does this mean for new antibiotics that are inspired by polymyxins? While the site of the bactericidal action of LPS is still debated^3,32,33^, it is clear that interaction with the OM plays a key role; PMBs either cause cell lysis by disruption of the OM, or they permeate across the OM to destabilize the inner membrane^32^. The mechanism of OM interaction we observed in our simulations points to the importance of both the acyl chain and the basic amino acids of PMBs. Consequently, these moieties would be important to incorporate into any alternative to PMB that is designed to interact with the OM *via* a similar mechanism. In PMBN, the sequence of events leading to insertion is not that same as PMB1, showing the impact of the tail of the latter. It has been shown by Vaara et al.^34^ that the DAB residues contribute to nephrotoxicity, and reduction of the number of these residues from 5 to 3 results reduces nephrotoxic effects and sensitizes Gram-negative bacteria to low concentrations of antibiotics. Nevertheless, we know that insertion requires replacement of the cations that cross-link LPS, thus alternatives to DAB that are positively charged could be a sensible strategy. We note that daptomycin is also a lipopeptide antibiotic with a fatty acid tail and charge of −3 at pH 7^35^, however it is only active against Gram-positive bacteria. This suggests that the lipopeptide approach is effective with the charges of the amino acids playing key roles.

It is worth reflecting on potential methodological limitations, and as is most often the case for MD simulations of biological systems, the biggest limitation is likely to be sampling. To mitigate for this as much as is pragmatic we have performed multiple simulations, used a validated model of ReLPS that allows for enhanced lateral diffusion^23^ within the outer leaflet of the OM. Furthermore, we have multiple copy numbers of OMPs and polymyxins within each simulation system providing many interaction and insertion events. We note here that single simulations of large systems such as viral surfaces which are composed of multiple copy numbers of a few protein types have previously been reported as allowing adequate sampling if each trajectory protein is treated as an individual trajectory^36^. In our study, the interaction of polymyxins, LPS, and OMPs precludes treating each OMP or PMB as an individual trajectory since our simulations involve multiple units within each system, and thus sampling is as extensive as is pragmatic with standard molecular dynamics.

In conclusion, we have revealed molecular-level details of the impact of PMBs on the *E. coli* OM and in doing so have also provided an explanation for AFM data of polymyxin-incubated OM organization. Additionally, we have provided compelling evidence for the mechanism *via* which PMBs penetrate into the hydrophobic core of the OM using the latest data on *E. coli* OM organization and have shown that the charges of the DAB residues as well as the presence of the acyl tail, play a role in insertion into the outer leaflet of the OM.

## Methods

### Coarse-grained molecular dynamics simulations

In our CGMD simulations, the proteins were coarse-grained using the martinize.py script as per the Martini 2.2 forcefield^37,38^, and the insane.py script^39^ was used to insert the OMPs into an OM model measuring 150 × 150 nm^2^. The OM model containing 218 OMPs arranged in a lattice was obtained from our previous simulation study on lipids mediating OMP assembly^18^. The OM model in this study consists of the ‘fast’ Re-level LPS^23^ in the outer leaflet, and phospholipids in the inner leaflet of the membrane in the ratio: 90% 16:0/18:1 palmitoyloleoyl-phosphatidylethanolamine (POPE), 5% 16:0/18:1 palmitoyloleoyl-phosphatidylglycerol (POPG), and 5% cardiolipin. Lipids were removed from the pores of the OMPs after usage of the insane.py script. A 3-dimensional layer of randomly distributed 1394 CG PMB1 molecules measuring 150 nm^2^ (XY plane) and ~4 nm (Z axis) was suspended above and parallel to the plane of the OM without contacting any OMPs. In addition, there was ~2.5 nm space along Z axis between the lowest PMB1 bead and the surface of the outer leaflet. The concentration of PMB1 molecules followed a 6:1 ratio of LPS:PMB1 following one of the proportions used in a previous study^14^. We also setup simulations containing PMBN which lack the fatty acid tail^40^. Therefore, we used the same setup and removed two Martini beads that corresponded to the fatty acid tails. The systems were solvated with Martini 2 water model and 0.15 M Na^+^ and Cl^−^ ion concentration, and finally neutralized with monovalent (Na^+^) cations. Divalent cations are not included while using the fast ReLPS model due to scaled-down charge-charge interactions between cations and anionic ReLPS beads.

Energy minimization was performed using the steepest descent integrator before and after solvating the system. It was ensured that the forces converged to less than 1000 kJ/mol/nm and less than 2000 kJ/mol/nm before and after solvation respectively. The system was then subjected to a step-wise NPT equilibration using the leap-frog integrator. The Berendsen barostat^41^ was used in a semi-isotropic setting to maintain the pressure at 1 bar with a compressibility of 3 × 10^−^^4 ^bar^−1^. First, the equilibration was run using a 10 fs time-step for a time-scale of 1 ns followed by using a 20 fs time-step for a time-scale of 30 ns. During equilibration, the PMB1 molecules were position-restrained with 20 kJ/mol/nm. The final snapshots from 3 equilibration simulations were used for 3 respective production simulations. During production, the Parrinello-Rahman barostat^42^ was used in a semi-isotropic setting with the compressibility maintained at 3 × 10^−4 ^bar^−1^. A flat-bottom potential of 1000 kJ/mol/nm up to 5 nm bi-directionally along the Z axis was applied to all PMB1 molecules to prevent them from passing through the periodic boundary and inserting into the inner leaflet of the membrane. For each system i.e., containing PMB1/ PMBN, all production simulation replicates were performed for 10 μs each. During equilibration and production, the van der Waals and coulombic interactions were both defined by a cut-off scheme at 1.1 nm along with a potential-shift modifier, and the temperature was maintained at 313 K using the velocity rescaling thermostat^43^.

### Atomistic molecular dynamics simulations

Atomistic molecular dynamics (ATMD) simulations were performed with the CHARMM36m forcefield^44^ using snapshots extracted from CGMD simulations containing PMB1 molecules. These snapshots were backmapped into atomistic resolution using CG2AT2^45^. Note that our CGMD simulations used the fast ReLPS model where the anionic beads were scaled down by 50% and used only monovalent cations. Therefore, before backmapping, the original Martini 2.2 ReLPS model was used, the system was neutralized by divalent cations (Ca^2+^), and energy minimized. The atomistic systems were subjected to energy minimization until the forces converged to 3000 kJ/mol/nm, followed by an NPT equilibration thrice for 5 ns each to obtain 3 distinct starting coordinates for production simulations. During equilibration, the protein backbone and PMB1 molecules were position restrained using a 1000 kJ/mol/nm force constant to maintain their conformation as observed at the end of the CGMD simulation. The equilibration utilized a timestep of 2 fs. The velocity rescaling thermostat^43^ was used to maintain the temperature at 313 K. The Berendsen barostat^41^ in a semi-isotropic setting was used to maintain the pressure at 1 bar with a compressibility of 4.5 × 10^−5 ^bar^−1^. The Particle Mesh Ewald (PME) algorithm was used to calculate long range electrostatics^46^ while the short range non-bonded (both coulombic and van der Waals) interactions used a cutoff of 1.2 nm. The LINCS algorithm was used to constrain hydrogen bonds^47^. The same protocol was used for production simulations except that the barostat and thermostat was changed to Parrinello-Rahman^42^ and Nosé-Hoover^48^, respectively. Production simulations were performed in 3 replicates over a timescale of 1000 ns each.

All ATMD and CGMD simulations were set up and run using Gromacs^49^ versions 2020.3 and 2022.4 respectively. Refer to Table 1 for details on all the simulations performed in this work.

**Table 1:** Details on the setup of all systems simulated in this work

System description	Resolution	Forcefield	Production timestep	Simulation replicates x time	Initial box size (nm^3^)
OM model with PMB1	Coarse-grained	Martini 2.2	20 fs	3 × 10 μs	150 × 150 x 30
OM model with PMBN	Coarse-grained	Martini 2.2	20 fs	3 × 10 μs	150 × 150 x 30
OM model without PMBs	Coarse-grained	Martini 2.2	20 fs	3 × 10 μs	150 × 150 x 30
Backmapped from CG sim1 containing PMB1 (4 OMPs)	atomistic	C36m	2 fs	3 × 1 μs	12.5 × 31.2 × 12.9
Backmapped from CG sim1 containing PMB1 (8 OMPs)	atomistic	C36m	2 fs	3 × 1 μs	32.3 × 19.8 × 15.3
Backmapped from CG sim2 containing PMB1 (8 OMPs)	atomistic	C36m	2 fs	3 × 1 μs	33.2 × 19.7 × 16
Backmapped from CG sim3 containing PMB1 (6 OMPs)	atomistic	C36m	2 fs	3 × 1 μs	19.9 × 28.2 × 17.9
Backmapped from CG sim3 containing PMB1 (6 OMPs)	atomistic	C36m	2 fs	3 × 1 μs	25.3 × 24.4 × 17.6

For all CGMD simulations performed with the Martini 2.2 forcefield, our fast ReLPS model was used^23^ where the net charge of the system was neutralized with monovalent cations. The net charge of all atomistic systems simulated using the CHARMM36m (C36m) forcefield were neutralized with divalent cations.

### Analysis and visualization

Gromacs tools were used to obtain near-neighbour distance distributions, number of contacts (*gmx mindist*), 2-dimensional densities (*gmx densmap*), and MSD (*gmx msd*). Lateral MSD (MSD_2d_) calculations were performed along 2 dimensions, i.e., along the plane of the membrane, and the center of mass motion was removed in all cases. The MSD_2d_ of OMPs was calculated using their backbone beads, and MSD_2d_ of lipids using their phosphate beads. Lateral diffusion coefficients (*D*_*2d*_) of OMPs and lipids were calculated using their averaged MSD_2d_ values (computed from 3 simulation replicates) using the Einstein relation: ‘*r*^*2*^*(t)* = *4Dt*’ which is applied to 2-dimensional systems^50^. Here, ‘*r*^*2*^*(t)*’ is the MSD for an elapsed period of time ‘*t*’, the factor 4 is a result of *2d* where ‘*d*’ is the number of dimensions = 2 (diffusion along the plane of the membrane), and ‘*D*’ is the diffusion coefficient. By differentiating w.r.t ‘*t*’ on both sides of the equation, we get: slope = 4*D*. The SciPy python package^51^ was used to perform linear regression and fit the slope to the averaged MSD_2d_ between 1.0 and 9.0 μs in the 10 μs simulations, after which the lateral diffusion coefficient was obtained by *D*_*2d*_ = slope/4.

The analysis of flow directionality of ReLPS and phospholipids was performed using the ‘2D streamplot’ package of the visualization module of MDAnalysis^52^ combined with NumPy^53^. All trajectories were considered from the 5 μs time point (with a time-step of 10 ns) for this analysis to ensure that both PMB types had associated with the OM by this time. A time-step of 10 ns was used, and only the ReLPS phosphates and OMP backbones were used for this analysis.

Image analysis was performed on the CGMD simulations to quantify the number of OMP-OMP, OMP-PMB, and OMP-PMB-OMP aggregates. This analysis leveraged OpenCV, an open-source python library for computer vision (opencv.org), along with the Python Imaging Library (PIL) for image processing. First, simulation frames from VMD OpenGL display were rendered in high resolution (8688 × 6552 pixels) in which OMPs and PMBs were assigned contrasting colours i.e., dark blue and white respectively. Then, these rendered image(s) were used to identify OMPs, PMBs and OMP-PMB aggregates as 2-dimensional objects, and assign distinct colours to each of them. To rule out PMB-PMB aggregates and quantify macromolecular aggregates (i.e., OMP-OMP, OMP-PMB, and OMP-PMB-OMP complexes) during image analysis, a minimum size (in pixels) was passed as a filter where PMB-PMB aggregates (smaller than OMP-OMP, OMP-PMB, OMP-PMB-OMP aggregates) were eliminated. In each frame, we also ruled out PMB molecules in solution and those far away from OMPs ( > 1.4 nm) to account for only those that associated with OMPs directly or indirectly. After filtering these molecules and assigning distinct colours to each macromolecular aggregate, the number of unique colours were counted using PIL to obtain the number of uncomplexed (distinct) OMP-PMB-OMP aggregates. Note that, due to the large number of distinct aggregates, many colours were used to be assigned to these aggregates. Therefore, some colours that are different (by hex colour code) may appear to be indistinguishable to the human eye.

OMP-PMB1-LPS-OMP and OMP-LPS-PMB1-LPS-OMP bridges were quantified as a function of time from the atomistic simulations using the MDAnalysis toolkit^52^, NumPy^53^, and Pandas^54^ python packages. This analysis was based on contacts defined by a distance cut-off of 0.4 nm (which includes van der Waals interactions, hydrogen-bonding, and short-range electrostatic interactions). First, we calculated the contacts of all PMB1 molecules with all OMPs and all ReLPS molecules contacting OMPs. The list of ReLPS molecules contacting OMPs was updated every frame. This data was obtained for each frame (1 ns) across all simulation replicates of each atomistic system and was used as raw data to then quantify OMP-PMB1-LPS-OMP and OMP-LPS-PMB1-LPS-OMP bridges. An OMP-PMB1-LPS-OMP bridge was identified when a PMB1 molecule simultaneously contacted an OMP and an ReLPS molecule which also contacted a different OMP. An OMP-LPS-PMB1-LPS-OMP bridge was identified when a PMB1 molecule simultaneously contacted two different ReLPS molecules, where each of these ReLPS molecules was in direct contact with two different OMPs. These rules were applied to every PMB1 molecule in the system in each simulation frame to obtain time-series data for all bridges forming and breaking dynamically during the simulations.

Distance cut-offs of 0.6 and 0.4 nm were used to define the contacts between molecules in CG and ATMD simulations respectively. The cut-off distance between the hydrogen atom and the acceptor in hydrogen bonds evaluated in atomistic simulations was 0.35 nm, and the cut-off for the hydrogen-donor-acceptor angle was 30°. Visual Molecular Dynamics (VMD)^55^ was used for visualization and molecular images where present in figures. The Matplotlib^56^ python package and Xmgrace (https://plasma-gate.weizmann.ac.il/Grace/) were used for generating plots.

### Statistics and reproducibility

Statistical analyses, i.e., computing averages and standard deviations (shown as shaded regions or error bars), were performed using 3 simulation replicates for each simulation system.

### Reporting summary

Further information on research design is available in the Nature Portfolio Reporting Summary linked to this article.

## Supplementary information

Supplementary material

Reporting Summary

## Data Availability

The simulation parameters used, the coordinates generated, and the numerical source data for each quantitative plot presented in this work are made available on Zenodo at 10.5281/zenodo.17438285^57^.
