# Supplementary material for: Polymyxins slow down lateral diffusion of proteins and lipopolysaccharide in the E. coli outer membrane
Source: Commun Biol. 2025 Dec 5;8:1751. doi: 10.1038/s42003-025-09161-x (PMC12680711; doi:10.1038/s42003-025-09161-x)
Supplement: Supplementary file 1 — Supplementary material [file 42003_2025_9161_MOESM1_ESM.pdf]

Supplementary Information for

Polymyxins slow down lateral diffusion of proteins and lipopolysaccharide in the *E. coli* outer membrane

Dheeraj Prakaash and Syma Khalid *

*Corresponding author. Email: syma.khalid@bioch.ox.ac.uk

Supplementary information includes:

- Figs. S1 to S20
- Supplementary references

Supplementary figures

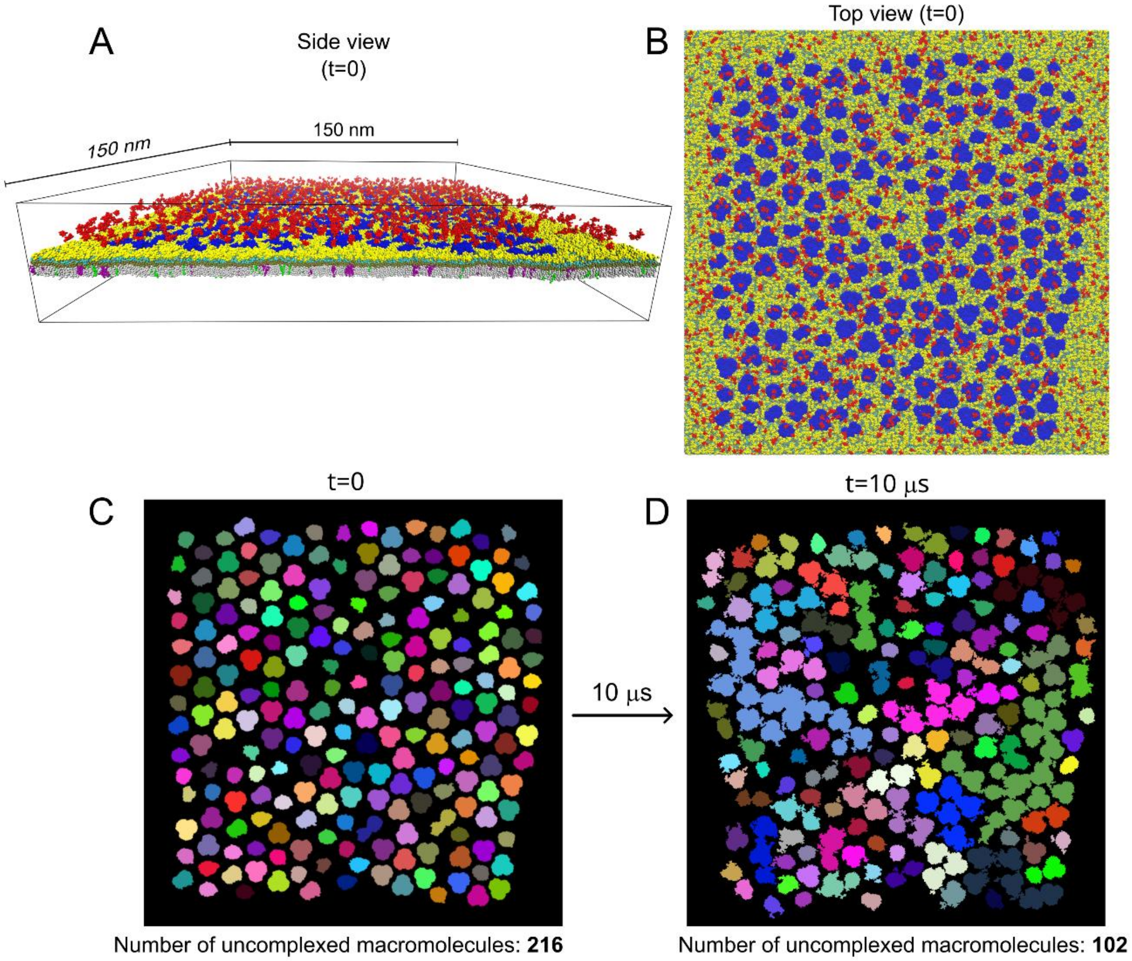

Fig. S1. Quantifying macromolecular aggregation using image analysis. (A) Side view and **(B)** top view of our model of an OMP-dense region containing PMB1 molecules at t=0. Colours assigned are: OMPs: blue, PMB1: red, ReLPS Kdo sugars: yellow, ReLPS sugar moieties in the lipid A region: cyan, ReLPS phosphate groups and fatty acid tails: brown, POPE: silver, POPG: green, cardiolipin: purple. **(C)** Image analysis being applied to the top-down view of our system at t=0 and **(D)** t=10 μs. Each individual OMP or individual OMP-OMP aggregate is assigned a different colour.

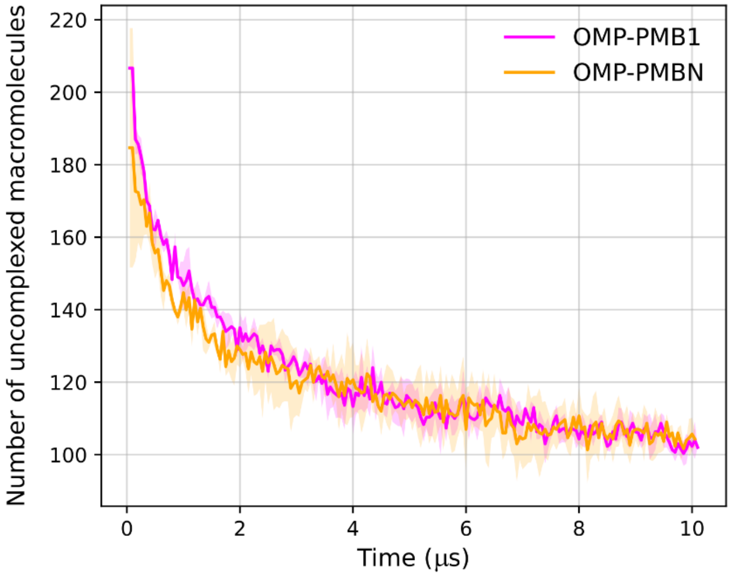

Fig. S2. Image analysis quantified to show the number of uncomplexed macromolecules vs time in CGMD simulations. Averages for both simulation systems i.e., containing PMB1 / PMBN are calculated from 3 simulation replicates.

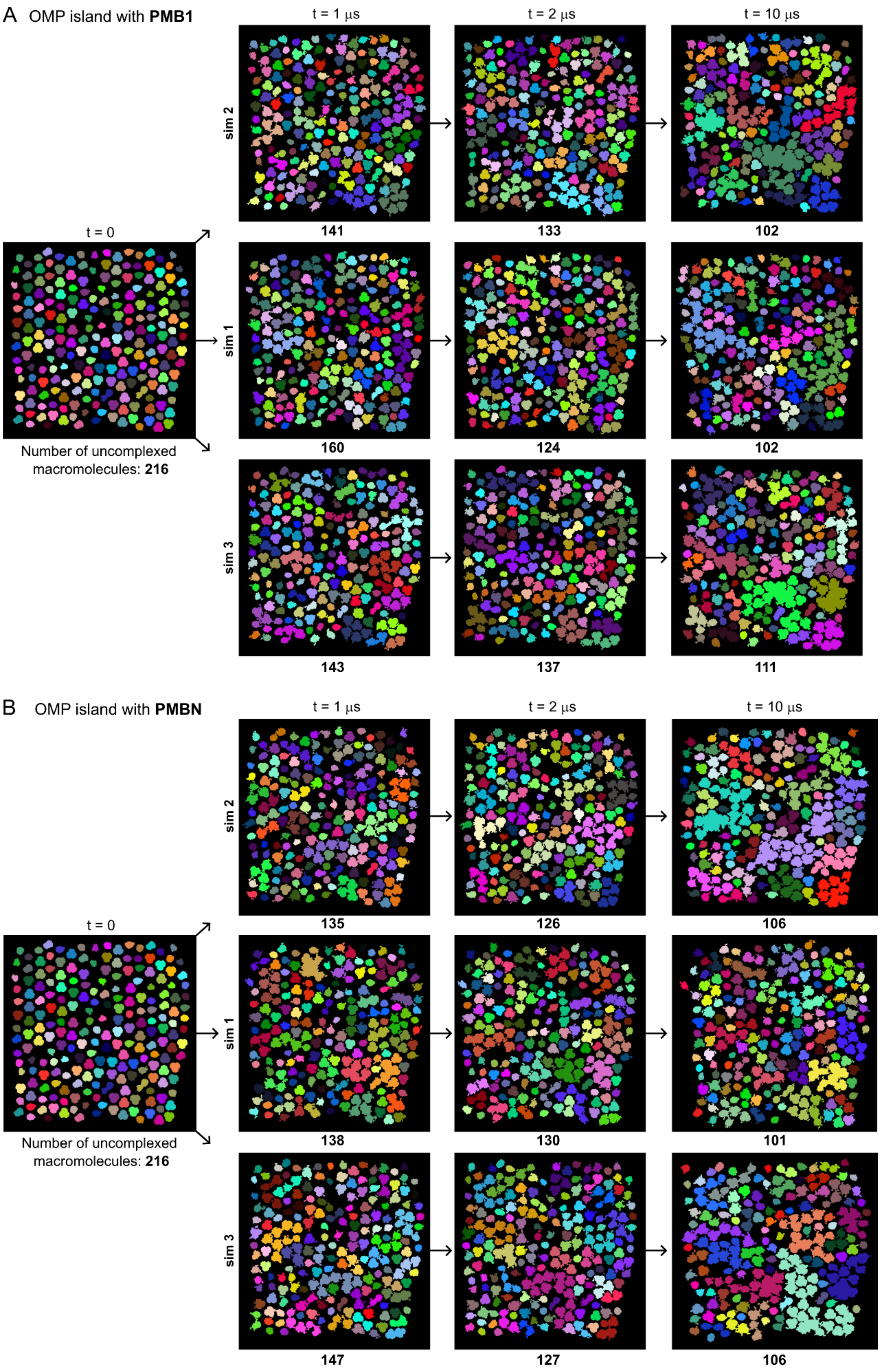
A OMP island with PMB1
t = 1 μs
t = 2 μs
t = 10 μs
t = 0
sim 2
sim 1
sim 3
141
133
102
160
124
102
143
137
111
Number of uncomplexed macromolecules: 216
B OMP island with PMBN
t = 1 μs
t = 2 μs
t = 10 μs
t = 0
sim 2
sim 1
sim 3
135
126
106
138
130
101
147
127
106
Number of uncomplexed macromolecules: 216

Fig. S3. Snapshots of image analysis obtained from both systems i.e., OM model associated with **(A)** PMB1 and **(B)** PMBN at simulation time (t)=0, 1 μs, 2 μs, and 10 μs. All images are viewed from the exterior region. The number of uncomplexed macromolecules is mentioned underneath the respective snapshot.

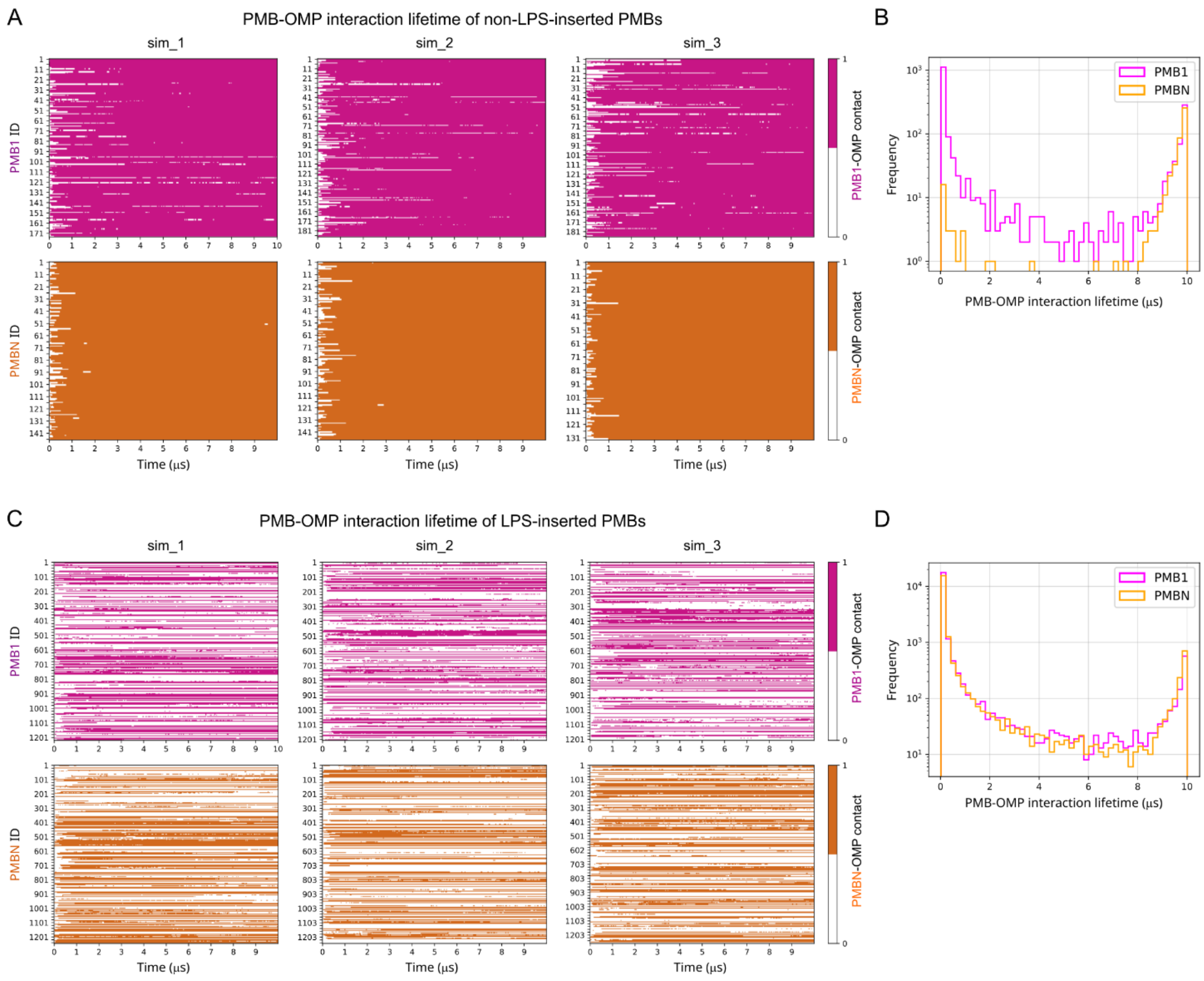

Fig. S4. PMB-OMP interaction lifetime. (A) Heatmaps obtained from each simulation replicate from both PMB1 and PMBN-containing CGMD simulations. Each cell in the heatmap indicates whether a particular PMB1 / PMBN molecule was in contact with an OMP (< 0.6 nm) or not at that point in simulation time. The time-points where it is and is not in contact with an OMP is represented with a colour and with white respectively. These data correspond to PMBs that interacted with OMPs but did not interact with ReLPS by the end of the (10 μs) simulation. **(B)** Histogram showing the distribution of PMB-OMP interaction lifetimes. This was calculated from the same data used for plotting (A). **(C)** The heatmaps were obtained as done for (A) except using data corresponding to PMBs that interacted with ReLPS by the end of the simulation. **(D)** Histogram showing the distribution of PMB-OMP interaction lifetimes as done for (B) but using data used in (C). Note that frequency of PMB-OMP interaction lifetimes (Y-axis) are on the log scale in (B) and (D).

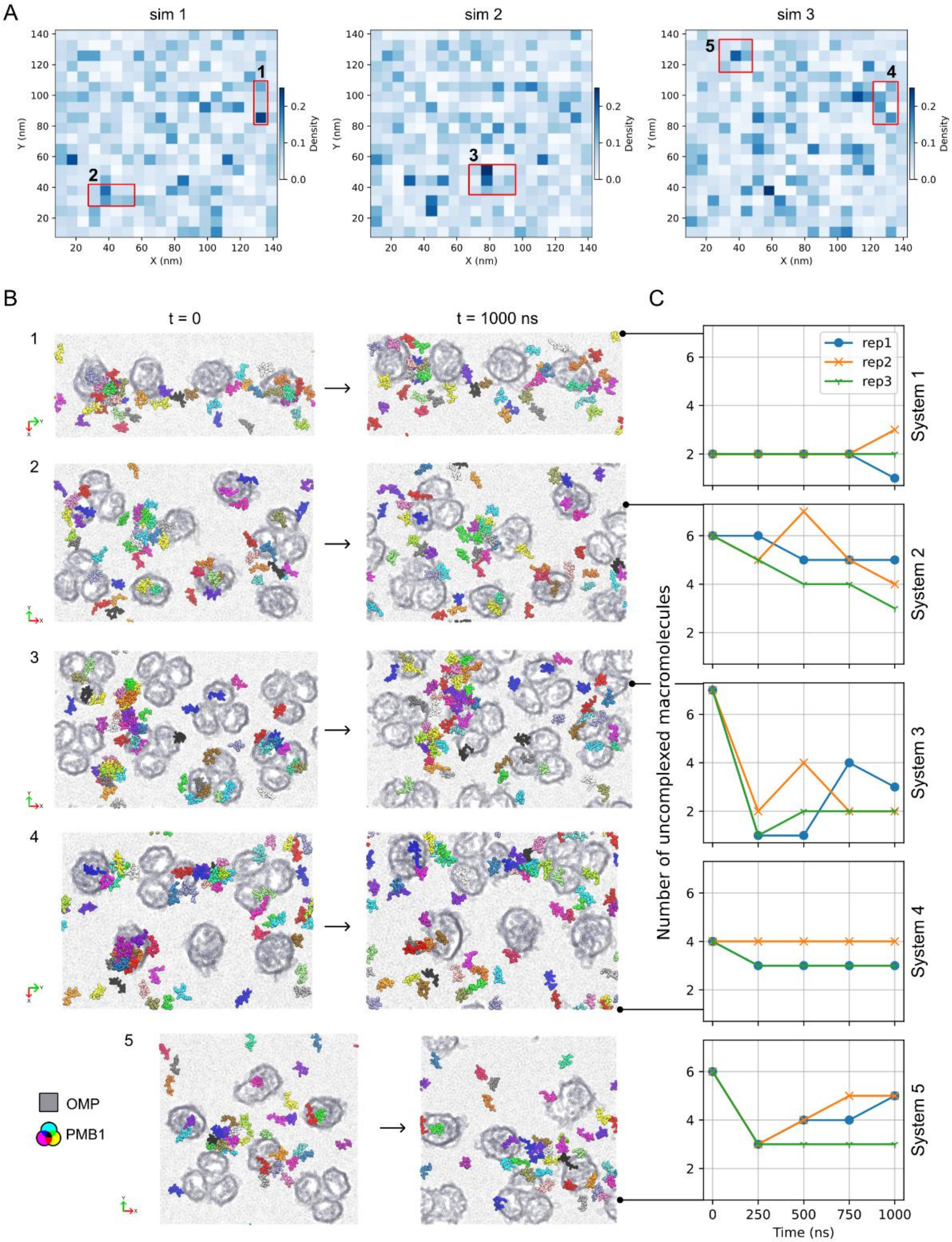
A
sim 1
sim 2
sim 3
X (nm)
Y (nm)
Density
B
t = 0
t = 1000 ns
OMP
PMB1
C
rep1
rep2
rep3
System 1
System 2
System 3
System 4
System 5
Number of uncomplexed macromolecules
Time (ns)

Fig. S5. Backmapping OMP-PMB1 bridges into atomistic resolution. (A) 2-dimensional density plots obtained from the 3 CGMD simulation replicates (at t = 2 μs) revealing with high density of PMB1 molecules. Backmapping these regions allowed sufficient sampling of PMB1 dynamics in atomistic simulations. **(B)** Snapshots from one of the atomistic simulation replicates at simulation time (t)=0 and 1 μs. **(C)** Change in the number of uncomplexed macromolecules vs time in 3 simulations of 5 atomistic systems. Black lines are drawn to indicate which system each plot is obtained from. Increase and decrease in the number of 'uncomplexed macromolecules' mean breaking and forming of OMP-OMP aggregates respectively.

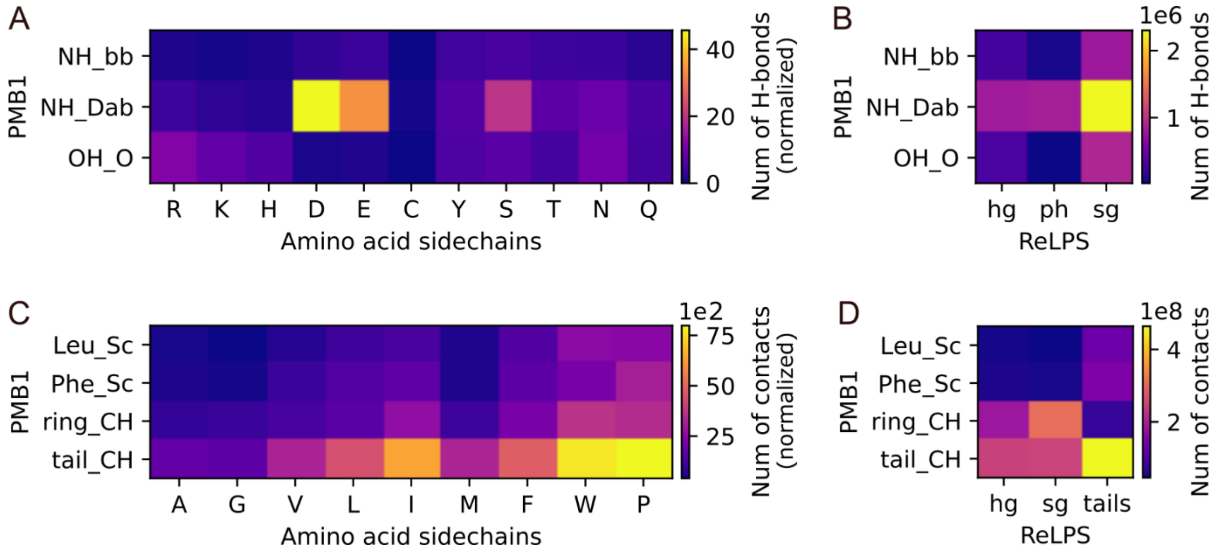

Fig. S6. Interaction details between PMB1, proteins, and ReLPS in atomistic simulations. **(A)** Number of hydrogen bonds (< 0.35 nm between donor hydrogen and acceptor) between different polar regions of PMB1 molecules (NH_bb: backbone NH groups, NH_Dab: sidechain NH groups of DAB residues, OH_O: hydroxyl or oxygen atoms) and the polar amino acid side chains. **(B)** Number of hydrogen bonds between these regions of PMB1 and specific parts of ReLPS (hg: headgroup excluding phosphates, ph: phosphate groups, sg: Kdo sugar moieties). **(C)** The number of contacts (< 0.4 nm) between the apolar regions of PMB1 and apolar amino acid side chains, and **(D)** between apolar regions of PMB1 and apolar components of ReLPS (CH groups in the headgroup, Kdo sugars, and ReLPS tails). To enable comparison of interactions between amino acid sidechains in (A) and (C), for example – to compare PMB1 interactions of aspartate to that of glutamate, normalization was performed by dividing the number of PMB1-amino acid interactions by the total number of those amino acids respectively. Data plotted in all heatmaps is averaged from 5 atomistic systems x 3 simulation replicates.

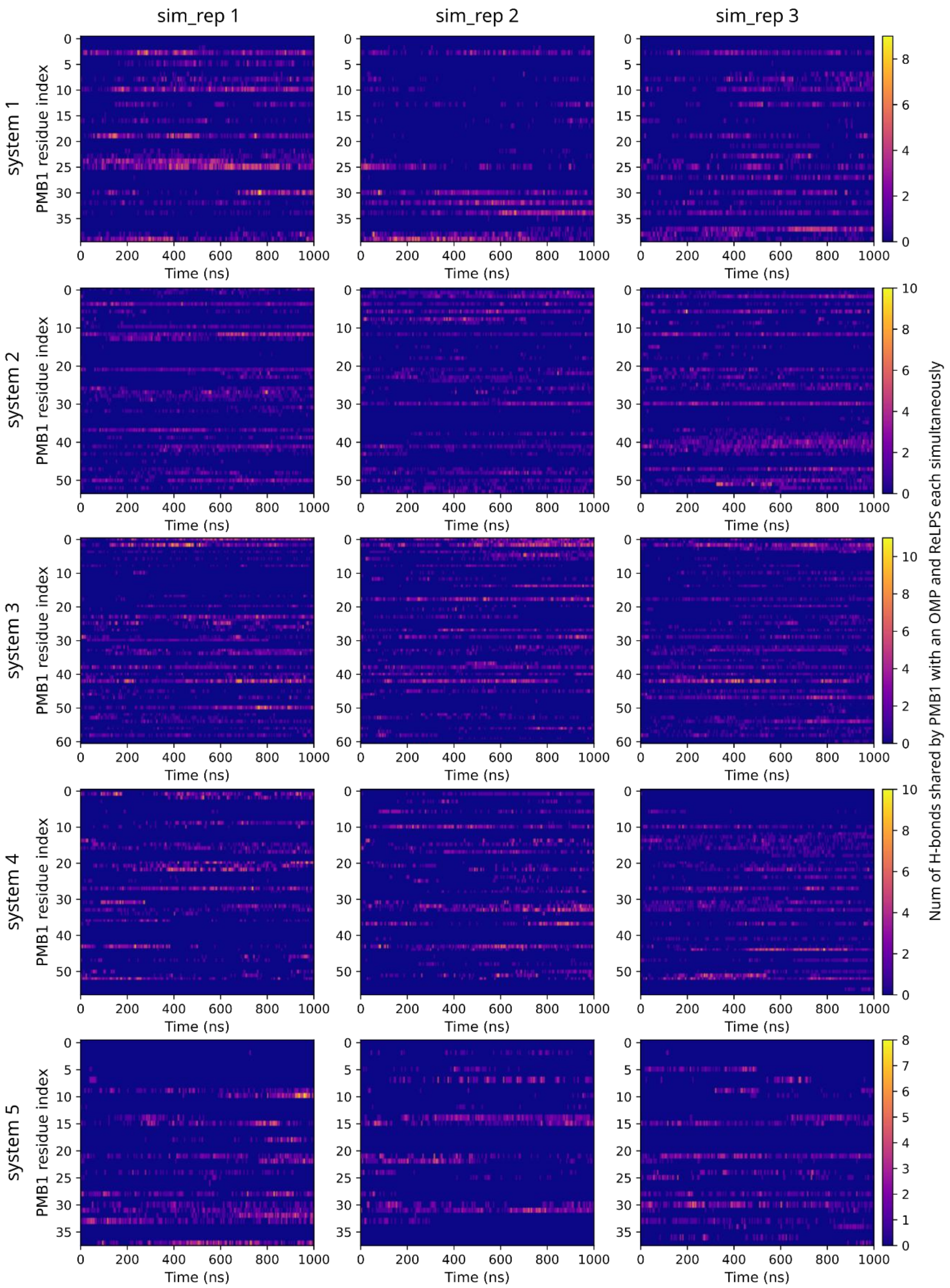

sim_rep 1
sim_rep 2
sim_rep 3
system 1
system 2
system 3
system 4
system 5
PMB1 residue index
Time (ns)
Num of H-bonds shared by PMB1 with an OMP and ReLPS each simultaneously

Fig. S7. Interactions of PMB1 with protein and ReLPS simultaneously vs time in atomistic simulations. Each cell in the heatmap represents the minimum number of hydrogen bonds simultaneously forming a PMB1-OMP interaction and a PMB1-ReLPS interaction in that simulation frame (dt=500 ps). For instance, if a PMB1 molecule forms 2 hydrogen bonds with an OMP and 3 hydrogen bonds with an ReLPS (or vice versa) at the same time, then the number assigned to that PMB1 molecule at that time frame is 2. These assigned numbers help identify: (i) which/ how many PMB1 molecules mediate 'strong' interactions between OMPs and ReLPS molecules i.e. more hydrogen bond pairs formed simultaneously between PMB1-OMP and between PMB1-ReLPS, and (ii) where PMB1 molecules mediate 'weak' interactions between OMPs and ReLPS molecules i.e. fewer hydrogen bond pairs formed simultaneously between PMB1-OMP and between PMB1-ReLPS.

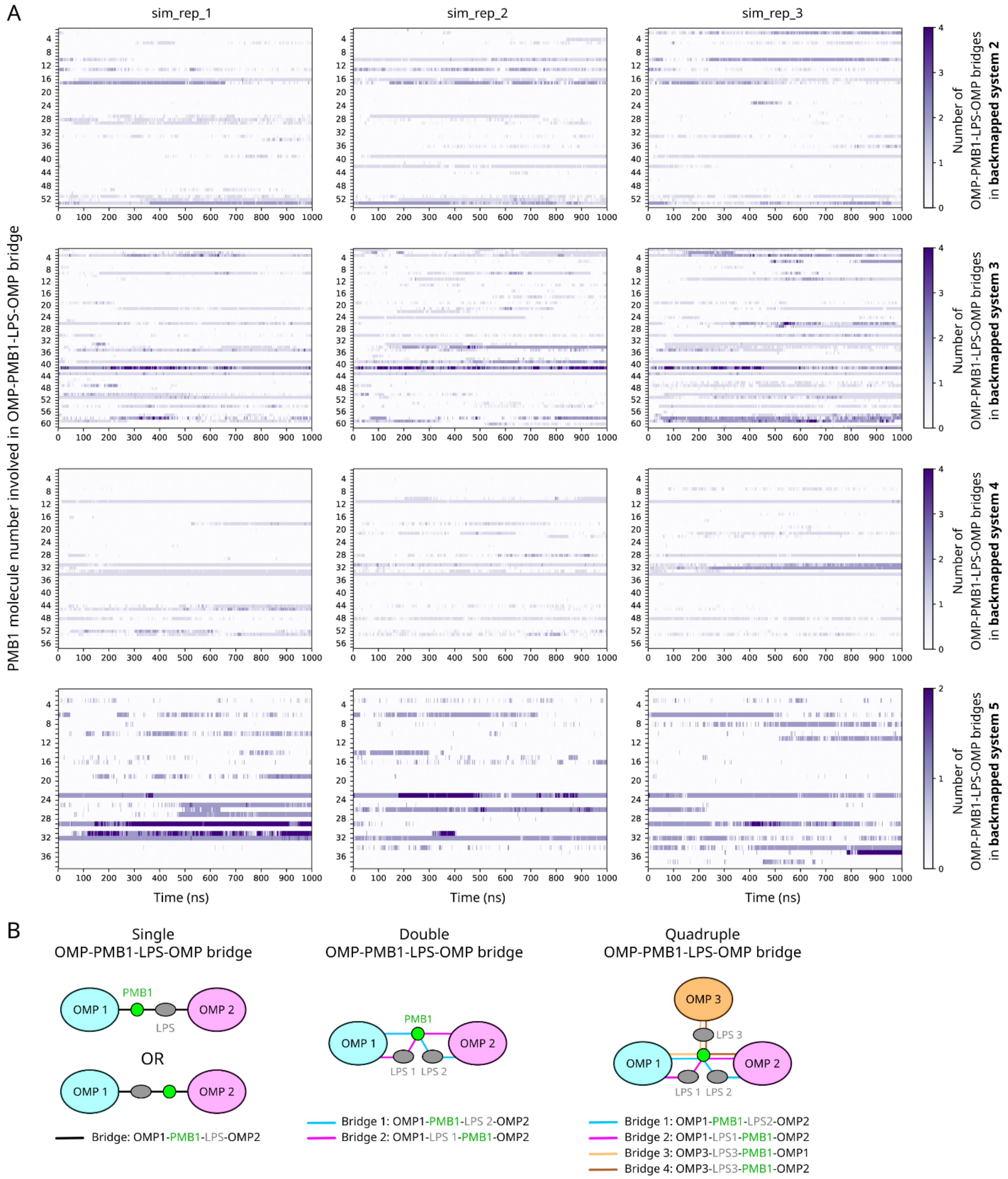

Fig. S8. OMP-PMB1-LPS-OMP bridges vs time. (A) Heatmap plots for each simulation replicate for atomistic systems 2-5. The heatmaps for atomistic system 1 are shown in **Fig. 3A**. The number of PMB1 molecules in each system differs and thus the Y-axis representing PMB1 molecule number differs for each row of plots. The data in each cell of the heatmap represents

the number of OMP-PMB1-LPS-OMP bridges. The maximum number of bridges is normalized across all 3 simulation replicates for each system, hence a single common colour bar per system. **(B)** Schematic illustrations of single, double and quadruple OMP-PMB1-LPS-OMP bridges (viewing from the extracellular region). Note that: (i) a single PMB1 cannot mediate 3 OMP-PMB1-LPS-OMP bridges logically; there can only be 1 or 2 bridges (formed between 2 OMPs) or 4 or 6 bridges (formed between 3 OMPs) mediated by a single PMB1, (ii) PMB1 directly contacts 2 OMPs (OMP-PMB1-OMP) in the double and quadruple bridges.

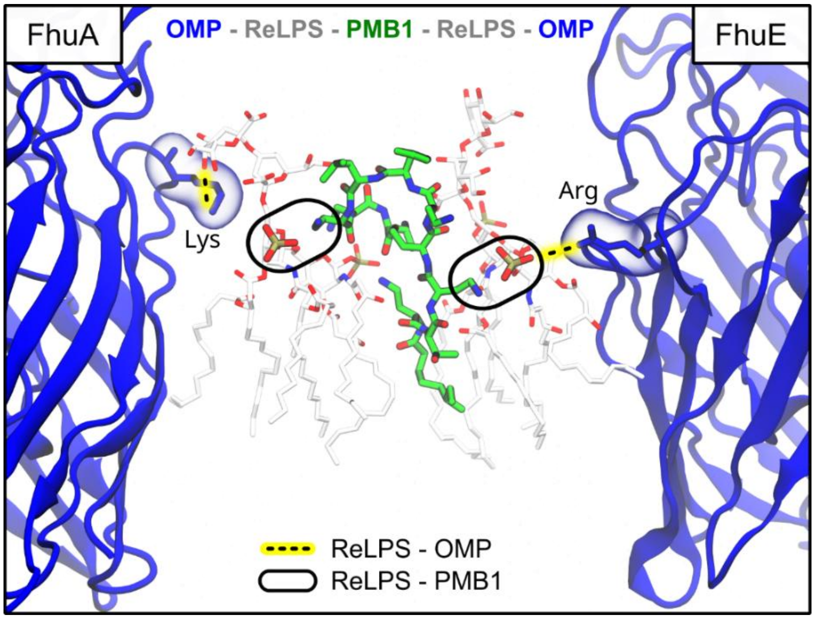

Fig. S9. Hydrogen bonding in an OMP-ReLPS-PMB1-ReLPS-OMP bridge. OMP backbones are shown in blue cartoon representation. The 2 ReLPS molecules in this bridge are shown as sticks (carbon atoms: white, oxygen: red, nitrogen: blue, phosphorus: brown). The PMB1 molecule interacting with both ReLPS molecules is shown as sticks as well (but its carbon atoms are green). The PMB1 is interacting with the phosphate groups of both ReLPS molecules *via* its DAB sidechains (shown within the oval shapes). The amino acid sidechains of the OMPs interacting with these ReLPS molecules are labelled and highlighted using surface representation. The lysine sidechain of FhuA is interacting with the Kdo region of one ReLPS (left), whereas the arginine sidechain of FhuE is interacting with the phosphate group of the other ReLPS (right). Other ReLPS molecules, phospholipids, water and ions are hidden for visual clarity. This snapshot was extracted from t=0 from one of the atomistic simulations containing 4 OMPs.

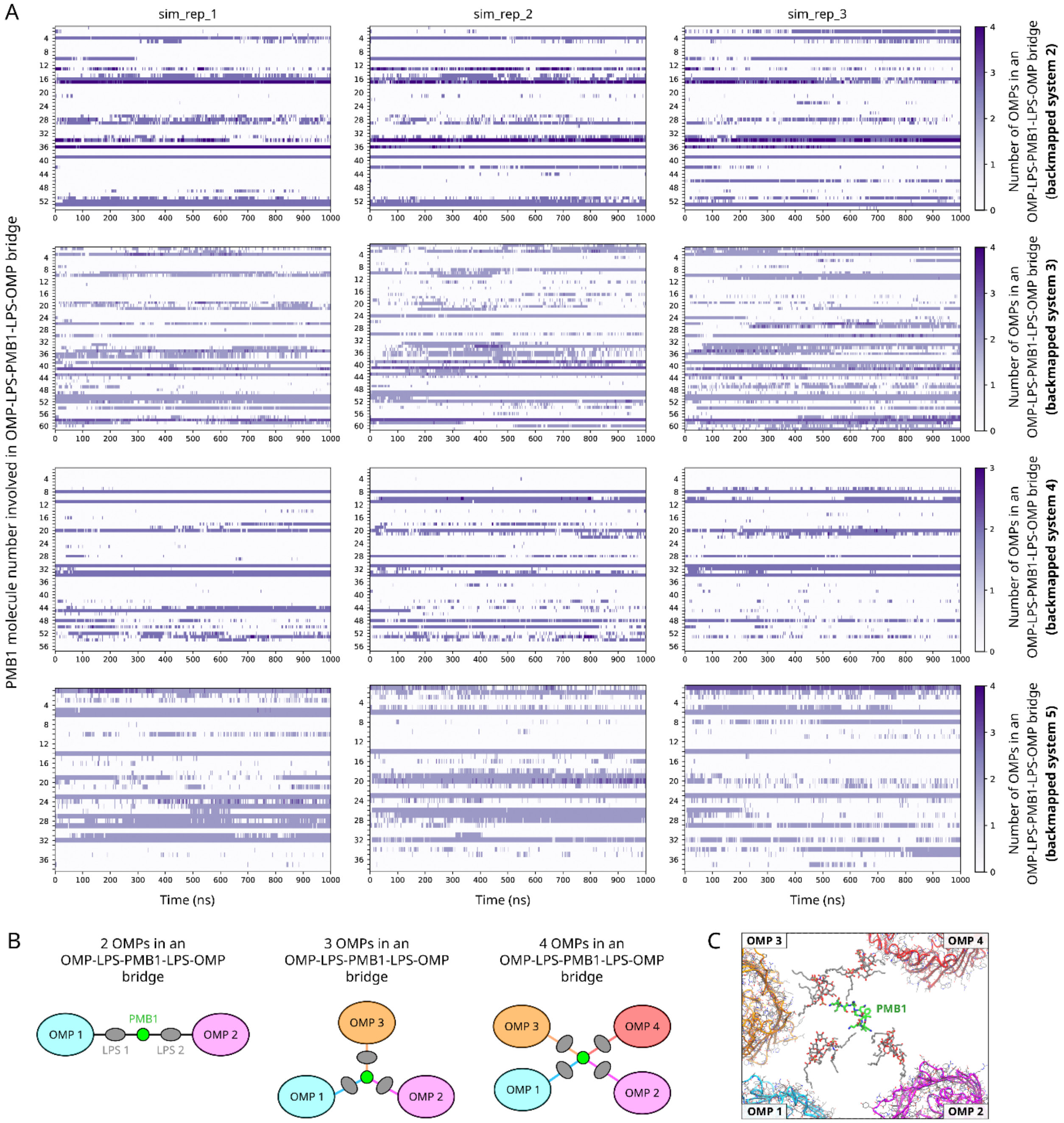

Fig. S10. OMP-LPS-PMB1-LPS-OMP bridges vs time. (A) Heatmap plots for each simulation replicate for atomistic systems 2-5. The heatmaps for atomistic system 1 is shown in Fig. 4D. The number of PMB1 molecules in each system differs and thus the Y-axis representing PMB1 molecule number differs for each row of plots. The data in each cell of the heatmap represent the number of OMPs in an OMP-LPS-PMB1-LPS-OMP bridge. Note that an OMP-LPS-PMB1-LPS-OMP bridge, by definition, cannot involve a single OMP; there must be at least 2.

Therefore, all data cells in the heatmaps represent values > 1. The maximum number of OMPs is normalized across all 3 simulation replicates for each system, hence a single common colour bar per system. **(B)** Schematic illustrations of a single PMB1 bridging 2, 3 and 4 OMPs *via* ReLPS molecules (viewing from the extracellular region). **(C)** A simulation snapshot (viewing from the extracellular region) showing a single PMB1 molecule bridging 4 OMPs each *via* a different ReLPS molecule. The PMB1 molecule is represented as green sticks, ReLPS molecules as gray sticks, OMP backbones as cartoons (coloured differently for each OMP), and OMP amino acid sidechains as lines.

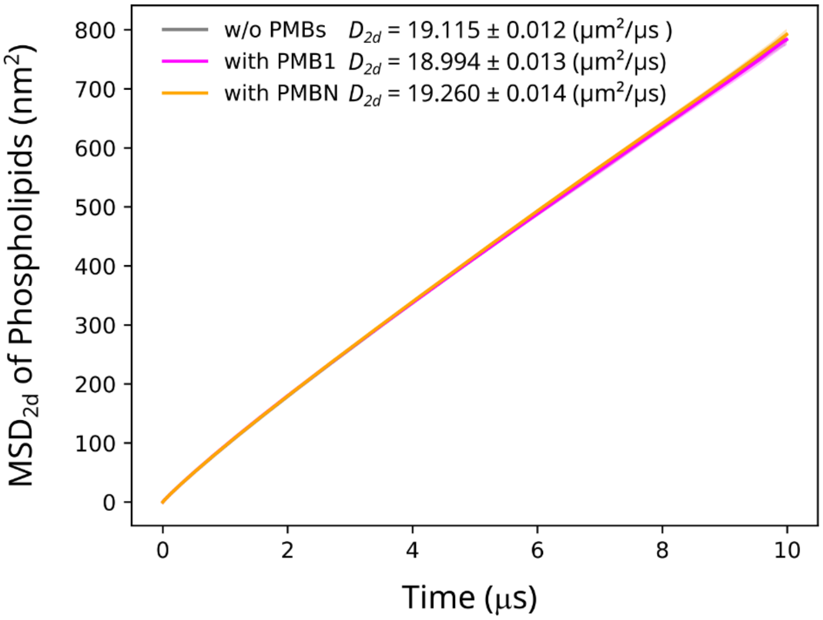

Fig. S11. Impact of PMB1 and PMBN on the lateral displacement of phospholipids in the inner leaflet of the OM in CGMD simulations. The average lateral mean squared displacement (MSD_{2d}) vs time of phospholipids are calculated from 3 simulation replicates (each for 3 systems) i.e. simulations containing (i) no PMB1 / PMBN (grey), (ii) PMB1 (magenta), and (iii) PMBN (orange). Averaged MSD_{2d} data are shown as lines while their standard deviations are shown as shaded regions of the same colour. The lateral diffusion coefficients (D_{2d}) calculated using the averaged MSD_{2d} values of each system are mentioned in the plot.

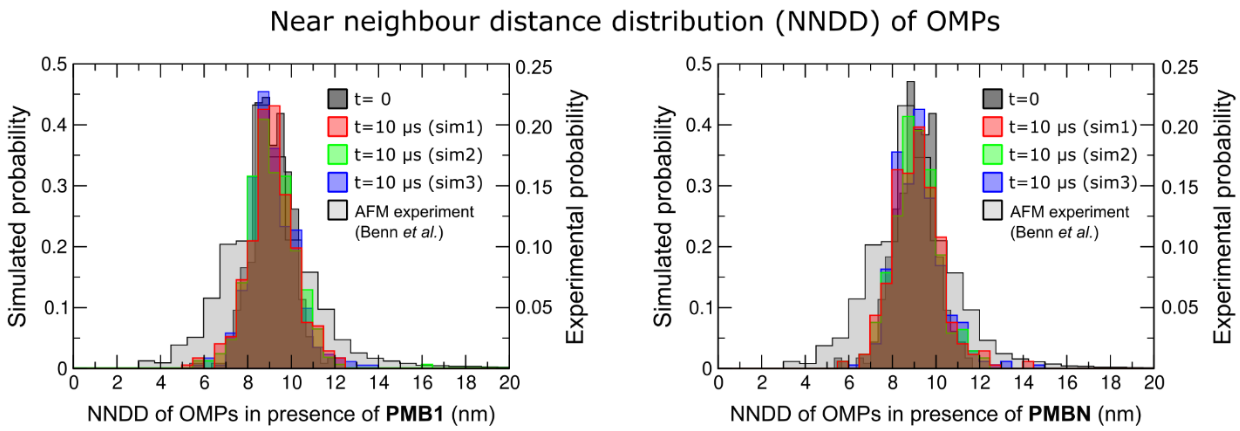

Fig. S12. Near neighbour distance distribution (NNDD) of OMPs. This data is obtained by measuring distances between the center of masses of 344 random pairs of OMP neighbours at t=0 and t=10 µs from 3 CGMD simulation replicates containing PMB1 (left) and PMBN molecules (right). These are compared to NNDD determined by AFM experiments [1] (ref (*19*) in main text).

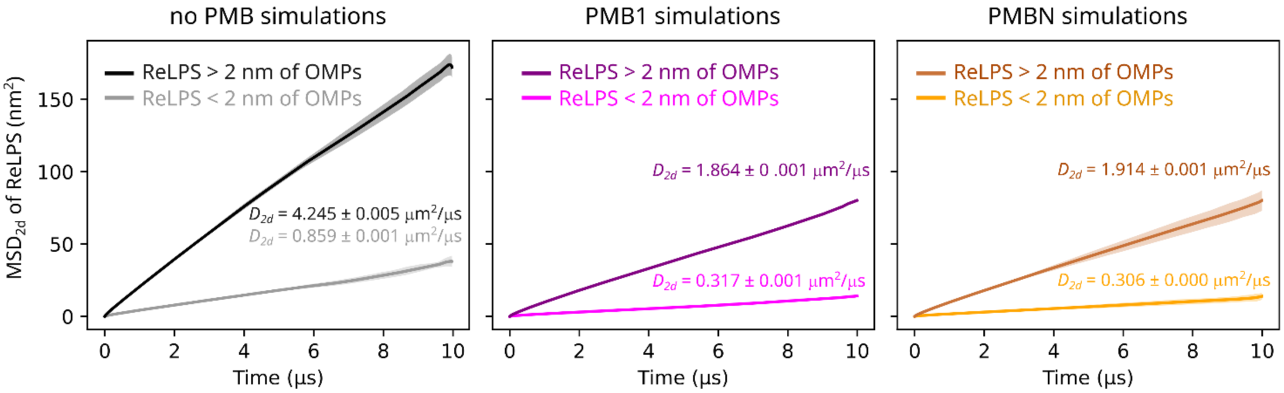

Fig. S13. Lateral diffusion regime of ReLPS within and outside the OMP-dense region. MSD_{2d} data for ReLPS within the OMP-dense region (mostly found < 2 nm from an OMP surface) and outside the OMP-dense region (> 2 nm from OMP surfaces) are shown. The MSD_{2d} of these two sub-populations of ReLPS were obtained for each simulation system i.e. (i) no PMB, (ii) PMB1, (iii) PMBN simulations (left to right plots). Each MSD_{2d} curve is represented by a solid line denoting its average across 3 simulation replicates, and a shaded region denoting its standard deviation. The lateral diffusion coefficients (D_{2d}) for each averaged MSD_{2d} curve are mentioned using matching font colour.

A

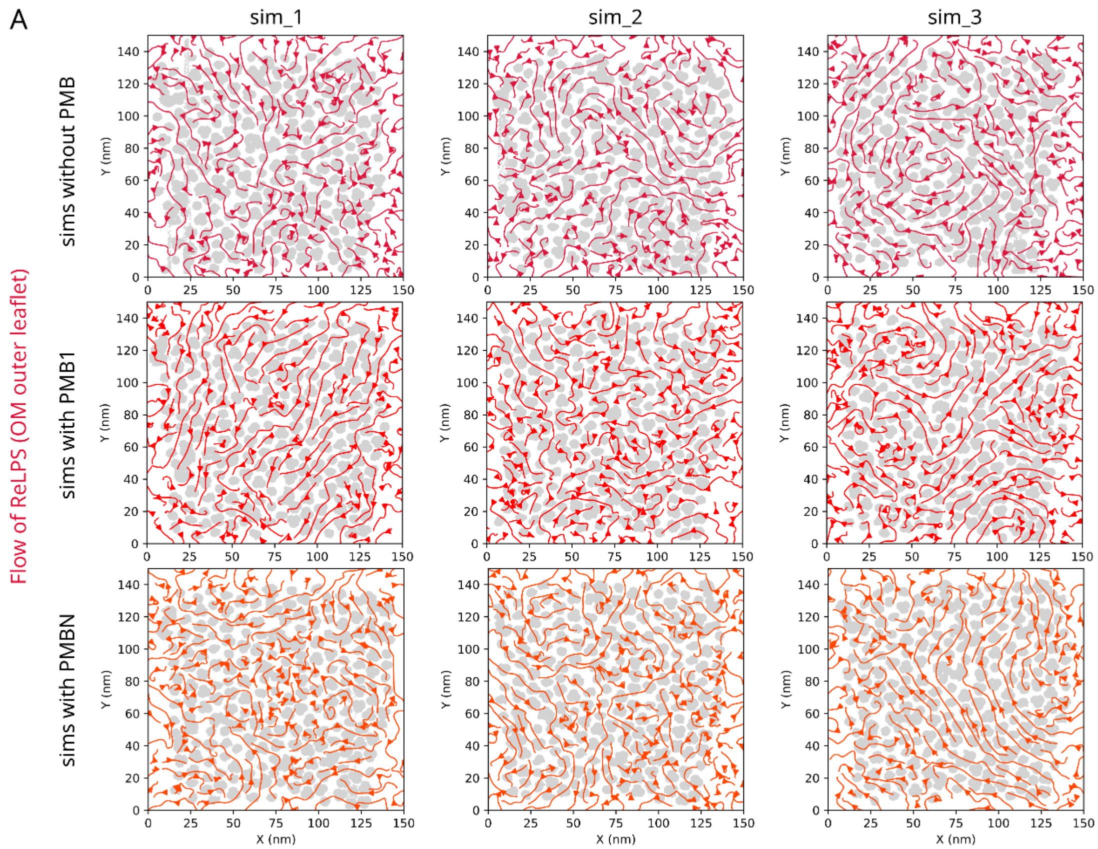

sim_1
sim_2
sim_3
sims without PMB
sims with PMB1
sims with PMBN
Flow of ReLPS (OM outer leaflet)
Y (nm)
X (nm)
0
25
50
75
100
125
150
0
20
40
60
80
100
120
140

B

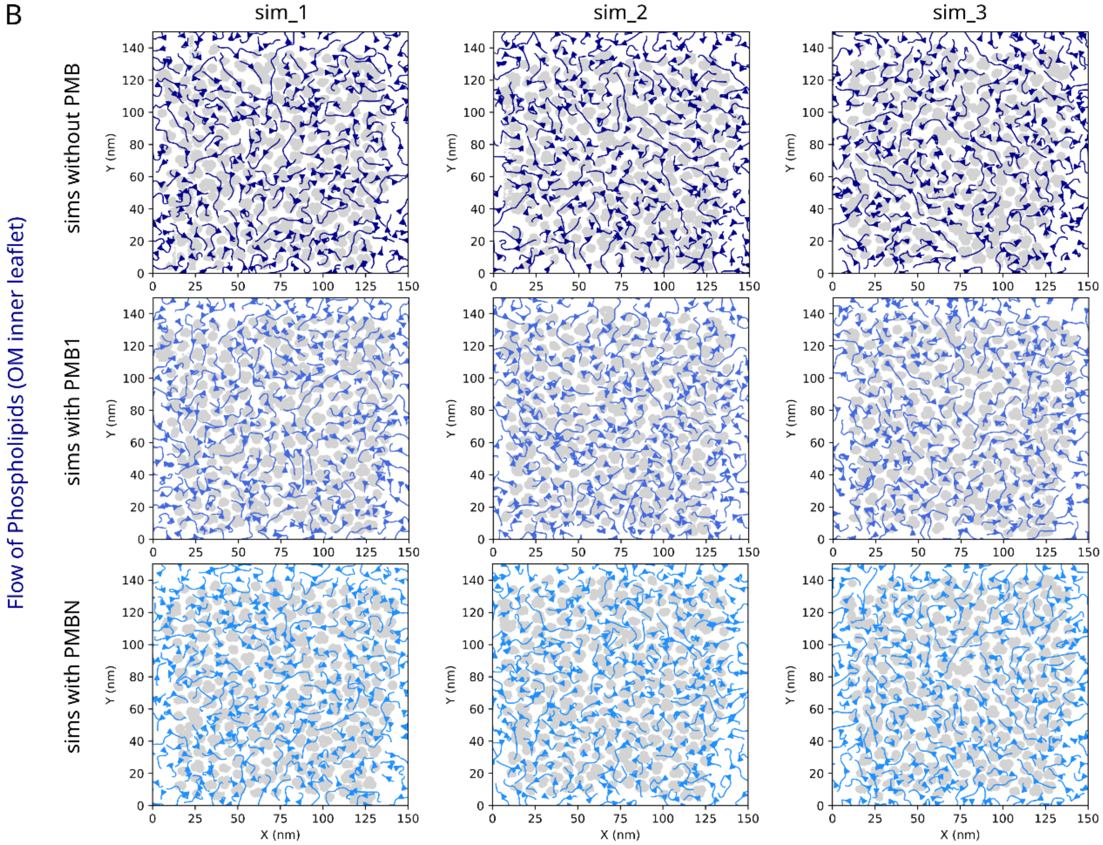

sim_1
sim_2
sim_3
sims without PMB
sims with PMB1
sims with PMBN
Flow of Phospholipids (OM inner leaflet)
Y (nm)
X (nm)
0
25
50
75
100
125
150
0
20
40
60
80
100
120
140

Fig. S14. Lateral flow directionality of ReLPS and phospholipids. (A) Flow of ReLPS (flow lines coloured in shades of red) and **(B)** phospholipids (flow lines coloured in shades of blue) shown separately for all 3 simulation replicates (5 – 10 μs) in 3 systems i.e. simulations with no PMB, with PMB1, and with PMBN. The X and Y axes in each plot represent the dimensions of the simulation system. The averaged coordinates of OMP backbones calculated are shown as light gray patches in the background in each plot for reference.

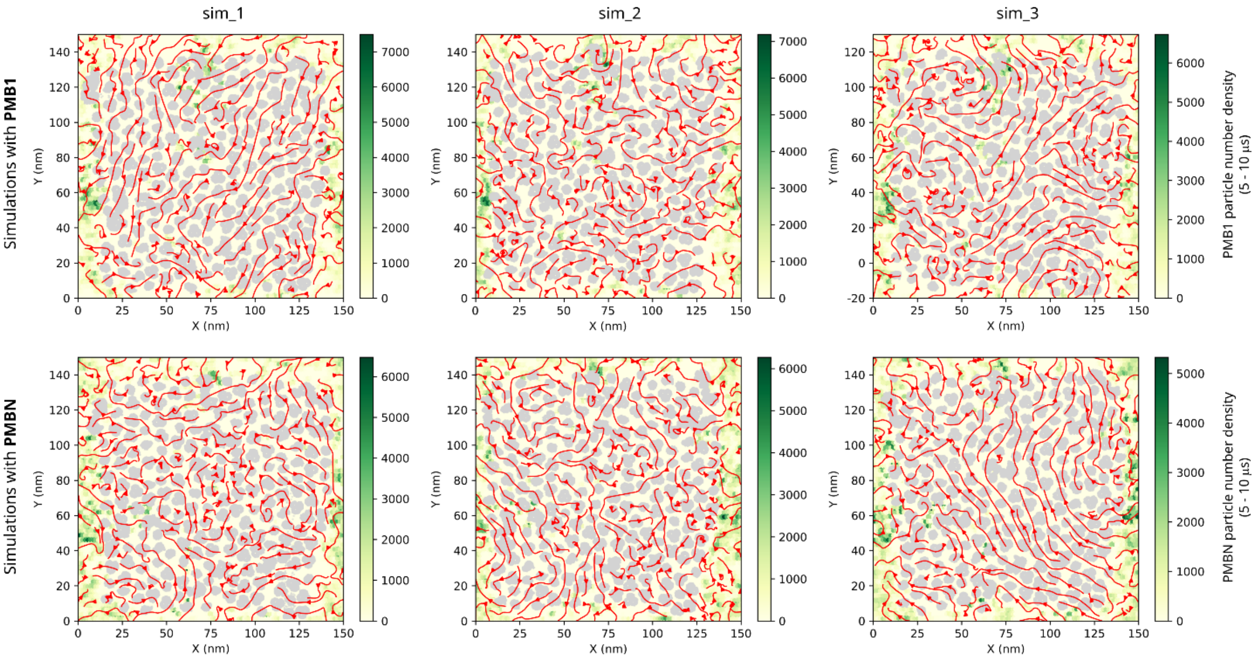

Fig. S15. Lateral flow directionality of ReLPS compared with the spatial density of PMBs that interacted directly with ReLPS. Plots in each column are generated from 3 separate simulation replicates starting from the 5 µs time point. The flow of ReLPS is shown as red lines and the averaged coordinates of OMP backbones are shown as light gray patches. The spatial density of PMB1 (top row) and PMBN (bottom row) is shown as a yellow-to-green (low-high) gradient. The X and Y axes in each plot represent the dimensions of the simulation system.

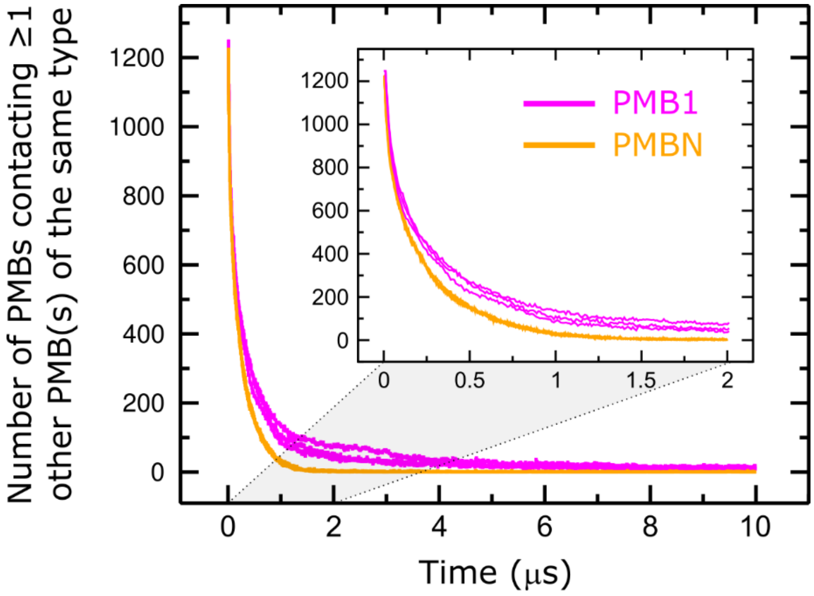

Fig. S16. PMB1 and PMBN aggregation vs time in CGMD simulations. The number of PMB1/PMBN molecules that contacted at least one other PMB molecule of the same type was computed per simulation frame. This data is plotted as a function of time for 3 simulation replicates separately for each system i.e. containing PMB1 and PMBN.

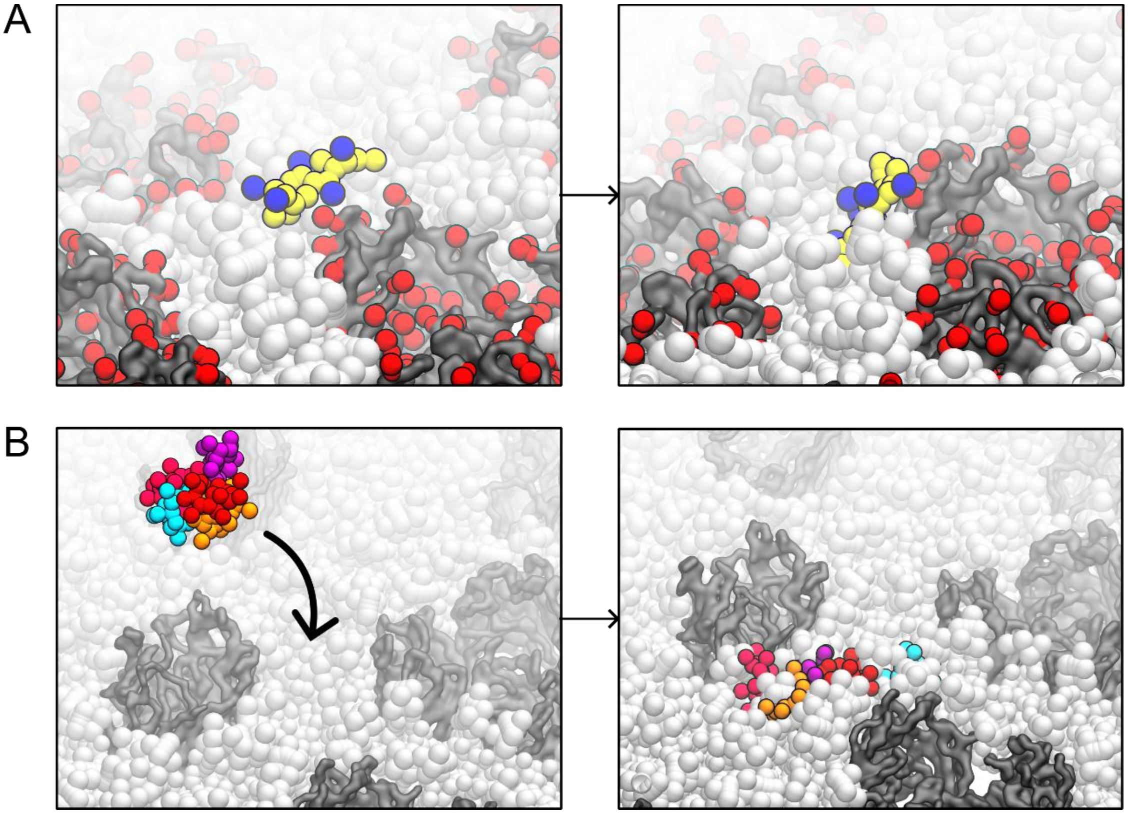

Fig. S17. Insertion of PMBs into the OM in CGMD simulations. (A) DAB residues of PMB1 first interact with acidic amino acids of OMPs before interacting with ReLPS molecules. **(B)** PMB1 molecules in the form of an aggregate in solution interacts directly with ReLPS molecules and dissociates to individual molecules.

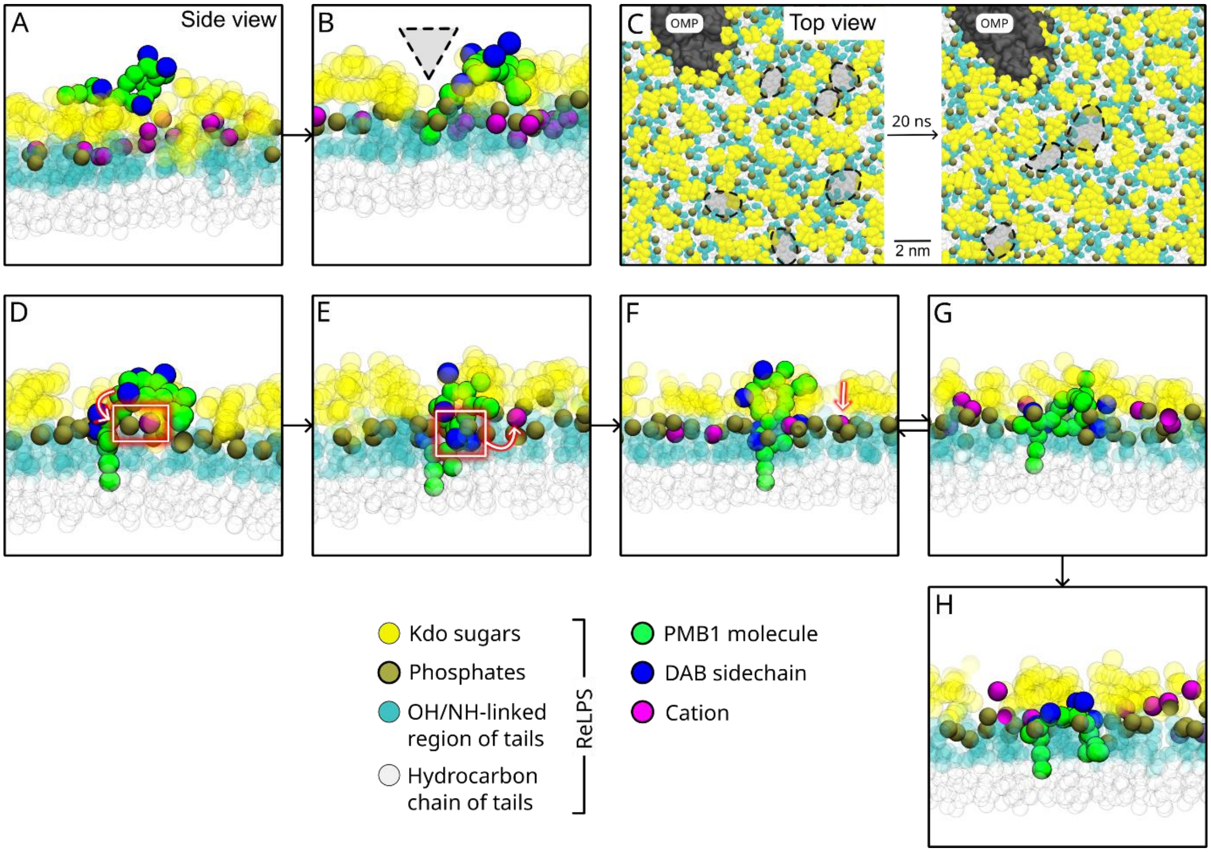

Fig. S18. Insertion mechanism of PMB1 into the ReLPS leaflet. (A) PMB1 interacts initially only with the Kdo sugar moieties of ReLPS molecules. **(B)** The fatty acid tail of PMB1 inserts into the hydrophobic region of the OM. **(C)** Top view snapshots (20 ns apart) of a subsection of the OMP-dense region showing the dynamic changes in the areas (shaded in gray) where PMBs can insert their fatty acid tails or hydrophobic amino acid sidechains. PMBs are hidden for visual clarity. **(D)** A phosphate headgroup of an ReLPS molecule interacting with a cation. **(E)** One of the PMB1 DAB residue sidechains replaces the cation to interact with the phosphate headgroup. In (D) and (E), other cations are hidden for visual clarity. **(F)** The elongated conformation, **(G)** L-shaped conformation, and **(H)** inverted U-shape conformation of the PMB1 molecule. Note that the double-sided arrow between (F) and (G) panels indicates that the PMB1 molecule can switch between these ('elongated' and 'L-shaped') conformations. The cation replaced by a DAB sidechain in (E) interacts with a phosphate group of a different ReLPS molecule in (F).

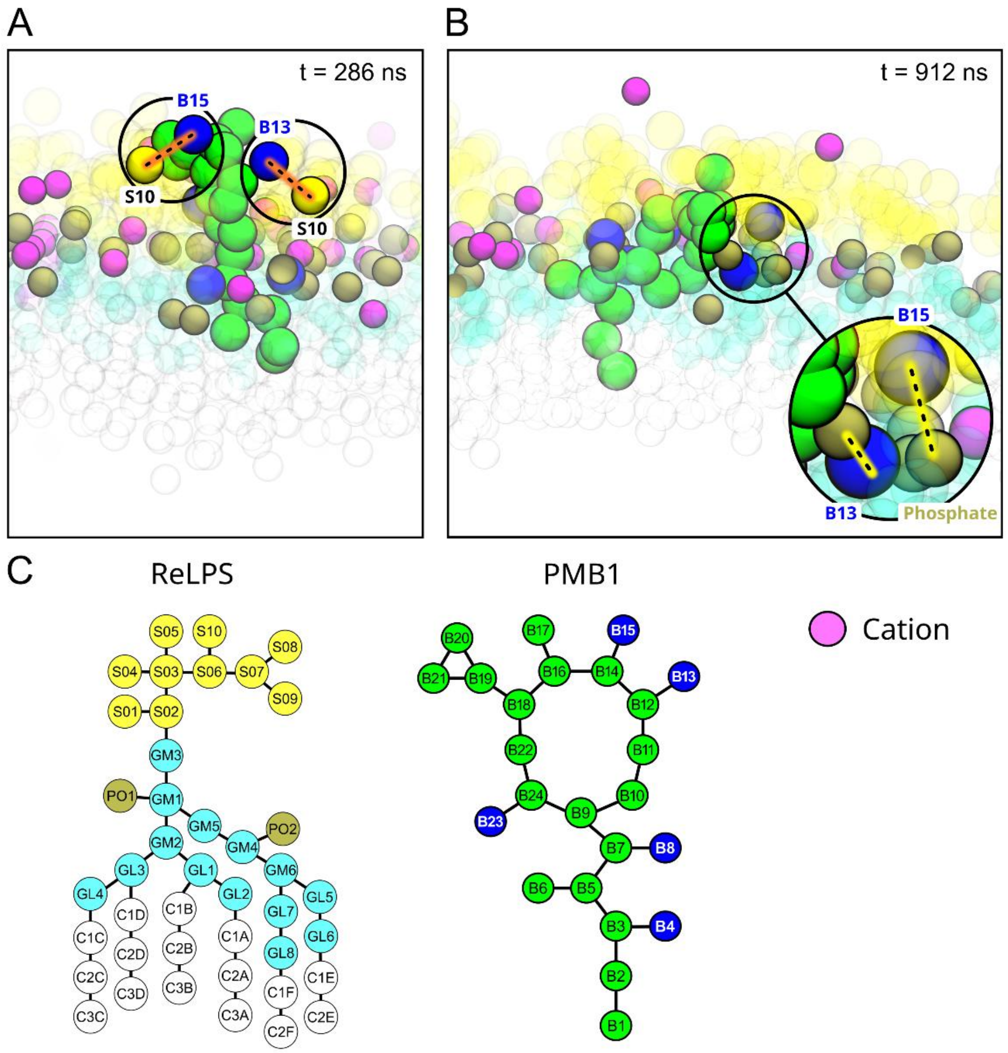

Fig. S19. The elongated and L-shaped conformations of PMB1 upon insertion into the ReLPS leaflet of the OM in CGMD simulations. (A) The elongated conformation of PMB1 is promoted by interactions of its DAB sidechain beads (B13 and B15) with anionic beads (S10; shown in yellow) in the Kdo region of 2 distinct ReLPS molecules. The whole ReLPS molecules of which these S10 beads are part of are hidden for visual clarity. **(B)** The L-shaped conformation of PMB1 is promoted by the interactions of its DAB sidechain beads (B13 and B15) with phosphate groups of ReLPS molecules. **(C)** 2-dimensional representations of the topology of coarse-grained (Martini 2) ReLPS and PMB1 following the same colour scheme used in (A) and (B). These are shown to help visualize the topological locations of the S10 bead of ReLPS and the B13/B15 beads of PMB1 as labelled in (A) and (B). Cations are represented by magenta spheres in (A) and (B).

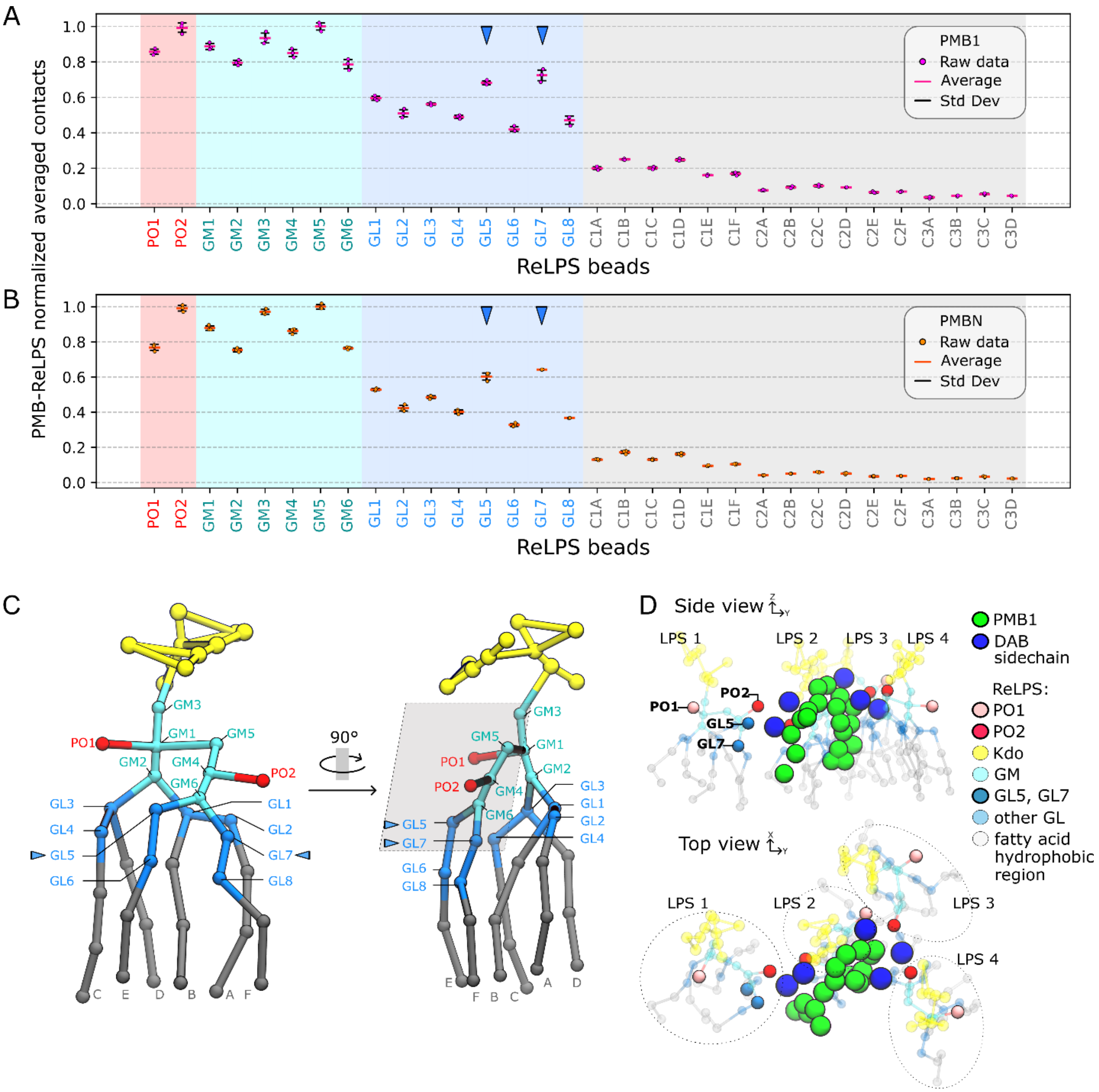

Fig. S20. Interactions of PMB1 and PMBN with the lipid A region of ReLPS. (A) Normalized averaged contacts of whole PMB1 and **(B)** PMBN molecules inserted into the membrane with each bead of the lipid A region all ReLPS molecules at t=2 μs. The red, cyan, blue and grey highlighted regions represent the phosphates, GlcN sugar moieties, OH/NH-linked regions of fatty acid tails, and hydrophobic region of fatty acid tails, respectively. Averages (coloured lines) and standard deviations (black error bars) of normalized contacts are calculated from 3 replicates for each simulation system. Normalization was performed by dividing all average contacts by the highest number of average contacts within the respective systems,

thereby obtaining a scale of 0 to 1. In (A), the value of 1 on the Y axis corresponds to 5494.3 before normalization, and in (B), it corresponds to 6351. The individual data points (from 3 simulation replicates) are shown as coloured dots overlaying their average and standard deviations. **(C)** Snapshots of a single coarse-grained (Martini 2) ReLPS molecule from 2 angles. The highlighted quadrilateral region indicates the preferred interaction site of PMB1/PMBN molecules according to data in (A) and (B). The PO, GM, GL beads of ReLPS, and the alphabets assigned to its fatty acid tails are labelled as described in the Martini 2.2 forcefield. In (A), (B), and (C), arrows point to GL5 and GL7 beads which form greater number of interactions with PMB1/PMBN molecules compared to other GL beads. **(D)** Side and top (where top is the cell exterior side) views of a snapshot of a membrane-inserted PMB1 molecule (represented as spheres) forming electrostatic interactions *via* its DAB sidechains with 4 ReLPS molecules (represented as transparent sticks) at t=2 μs. The DAB sidechains interact with the PO2 beads of all 4 ReLPS molecules and with PO1 beads of 2 out of 4 ReLPS molecules. In the top view, the GL5, GL7 and PO beads of LPS 1 are labelled to show that GL5 and GL7 are the closest to PO2 and thus PMB1 interacts more with GL5 and GL7 compared to other GL beads.

Supplementary references

1. Benn, G. *et al.* Phase separation in the outer membrane of *Escherichia coli*. *Proc. Natl. Acad. Sci. U.S.A.* **118**, e2112237118 (2021).
